# Hierarchical patterning modes orchestrate hair follicle morphogenesis

**DOI:** 10.1371/journal.pbio.2002117

**Published:** 2017-07-11

**Authors:** James D. Glover, Kirsty L. Wells, Franziska Matthäus, Kevin J. Painter, William Ho, Jon Riddell, Jeanette A. Johansson, Matthew J. Ford, Colin A. B. Jahoda, Vaclav Klika, Richard L. Mort, Denis J. Headon

**Affiliations:** 1 The Roslin Institute and Royal (Dick) School of Veterinary Studies, University of Edinburgh, Edinburgh, United Kingdom; 2 FIAS and Faculty of Biological Sciences, University of Frankfurt, Germany; 3 School of Mathematical & Computer Sciences, Heriot-Watt University, Edinburgh, United Kingdom; 4 Cancer Research UK Edinburgh Centre and MRC Human Genetics Unit, Institute of Molecular Medicine, Western General Hospital, University of Edinburgh, Edinburgh, United Kingdom; 5 School of Biological and Biomedical Sciences, Durham University, Durham, United Kingdom; 6 Department of Mathematics, Faculty of Nuclear Sciences and Physical Engineering, Czech Technical University in Prague, Prague, Czech Republic; 7 Division of Biomedical and Life Sciences, Faculty of Health and Medicine, Lancaster University, Bailrigg, Lancaster, United Kingdom; Lincolns Inn Fields Laboratory, United Kingdom of Great Britain and Northern Ireland

## Abstract

Two theories address the origin of repeating patterns, such as hair follicles, limb digits, and intestinal villi, during development. The Turing reaction–diffusion system posits that interacting diffusible signals produced by static cells first define a prepattern that then induces cell rearrangements to produce an anatomical structure. The second theory, that of mesenchymal self-organisation, proposes that mobile cells can form periodic patterns of cell aggregates directly, without reference to any prepattern. Early hair follicle development is characterised by the rapid appearance of periodic arrangements of altered gene expression in the epidermis and prominent clustering of the adjacent dermal mesenchymal cells. We assess the contributions and interplay between reaction–diffusion and mesenchymal self-organisation processes in hair follicle patterning, identifying a network of fibroblast growth factor (FGF), wingless-related integration site (WNT), and bone morphogenetic protein (BMP) signalling interactions capable of spontaneously producing a periodic pattern. Using time-lapse imaging, we find that mesenchymal cell condensation at hair follicles is locally directed by an epidermal prepattern. However, imposing this prepattern’s condition of high FGF and low BMP activity across the entire skin reveals a latent dermal capacity to undergo spatially patterned self-organisation in the absence of epithelial direction. This mesenchymal self-organisation relies on restricted transforming growth factor (TGF) β signalling, which serves to drive chemotactic mesenchymal patterning when reaction–diffusion patterning is suppressed, but, in normal conditions, facilitates cell movement to locally prepatterned sources of FGF. This work illustrates a hierarchy of periodic patterning modes operating in organogenesis.

## Author summary

Repeating anatomical units, forming a pattern, are present in different parts of the body. This is particularly obvious in the skin, where the many hair follicles become regularly arranged and fixed in place in the embryo as a pattern of evenly spaced spots. The basis for the formation of such repeating patterns has attracted a number of theoretical explanations. One influential theory is that cells first issue chemical signals to one another to produce a map of the pattern, which they then follow to assemble a structure, like a hair follicle. Alternative theories instead suggest that cells cluster together directly, without reference to a pre-existing map, to drive pattern formation. In this study, we find that a set of chemical signals known to be important for early hair follicle formation form a network capable of producing a patterned template to which cells then respond, moving according to its instructions. This supports the signal-based theory for pattern formation. Strikingly, though, by imposing the conditions of the incipient hair follicle evenly across the entire skin, we reveal that skin cells can also form patterns directly by coalescing, without requiring instructions from a pre-existing pattern. However, this ability of cells to pattern directly is normally subservient to their following of a quickly forming pattern template defined by chemical signals. This work highlights that different ways of making biological patterns can coexist in the same embryonic organ but that one is normally subordinated to the other.

## Introduction

**Fig 1:**
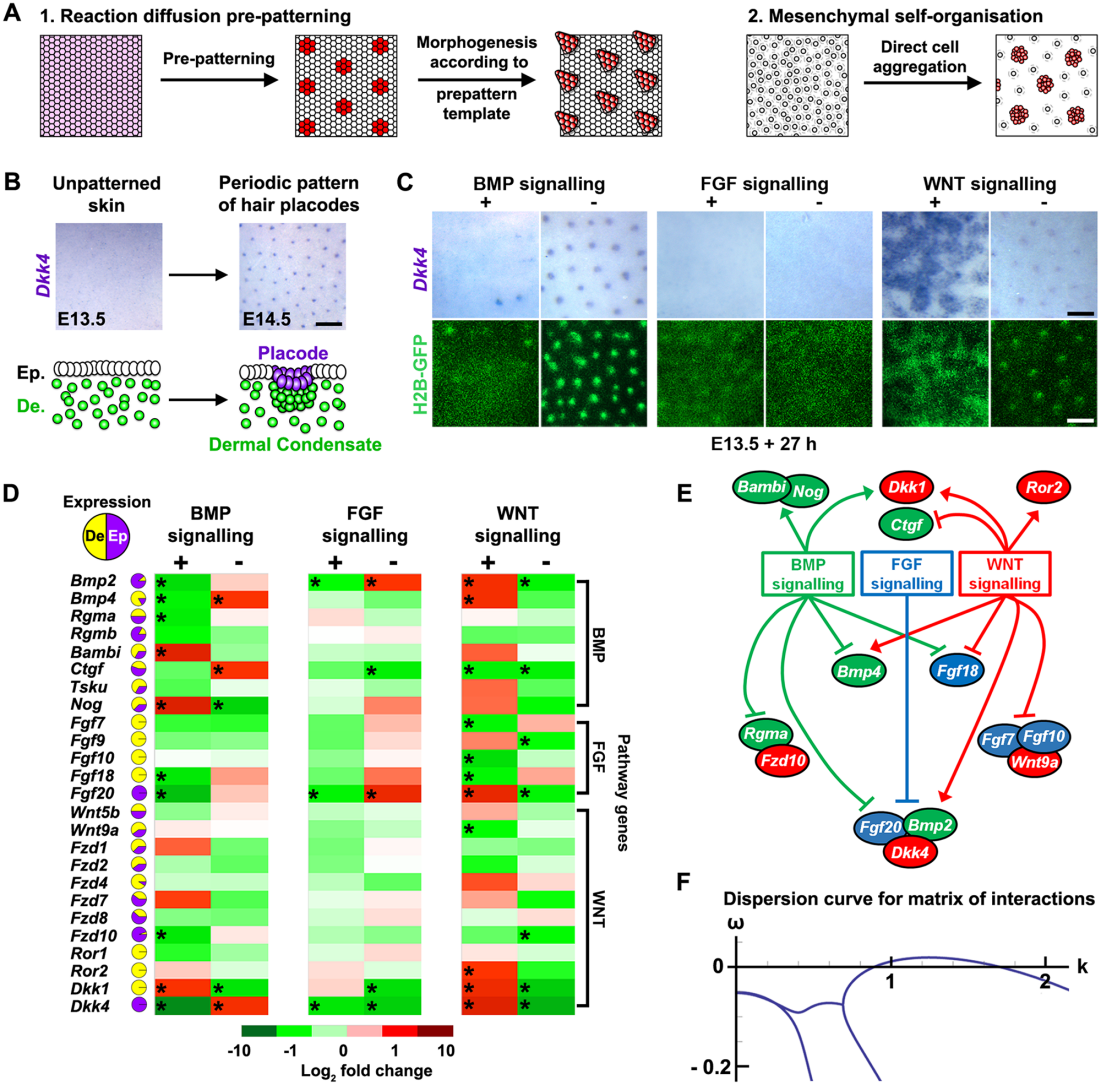
Interrelationships between BMP, FGF, and WNT signalling in skin patterning. (A) Schematic illustrating the 2 general theoretical models explaining the emergence of repeating patterns: Turing reaction–diffusion-driven systems and mesenchymal self-organisation. (B) Schematic of the process of hair follicle formation depicting acquisition of epidermal foci of *Dkk4* in placodes coupled with the underlying mesenchymal cell accumulation. (C) Pattern responses of skin to stimulation and repression of bone morphogenetic protein (BMP), fibroblast growth factor (FGF), and wingless-related integration site (WNT) pathways. Detection of the epidermal placode marker *Dkk4* and cell arrangement through green fluorescent protein (GFP) signal in the TCF/Lef::H2B-GFP line in E13.5 mouse dorsal skin explant cultures treated for 27 h with BMP4 (500 ng/ml), LDN193189 (BMP receptor inhibitor) (10 μM), FGF9 (1 μg/ml), SU5402 (FGFR inhibitor) (25 μM), CHIR99021 (GSK3 inhibitor to stimulate WNT/β-catenin signalling) (10 μM), or IWR-1 (WNT/β-catenin signalling inhibitor) (50 μM). Scale bars: 250 μm. (D) Heatmap depicting quantitative reverse transcription polymerase chain reaction (qRT-PCR) fold changes of candidate genes in response to 6 h stimulation or inhibition of the BMP, FGF, or WNT signalling pathways. Pie charts show the fraction of dermal (yellow) to epidermal (purple) expression of each gene detected in unstimulated skin (see S1 Table). Statistical significance from control skins was calculated using a Student *t* test (*p < 0.05 and > ± 1.8-fold change). The raw numerical data for the heatmap can be found in S1 Data. (E) Gene regulatory network derived from the transcriptional responses to BMP, FGF, or WNT pathway stimulation shown in (D). (F) Dispersion curves calculated from matrix of interactions (S3F Fig) show that Turing instability (curve breaking the x-axis) can be achieved by a regulatory network with this structure when components of each pathway can diffuse (see S1B and S1C Appendix for details).

Diverse structures, such as the skeletal elements of the limb, rugae of the palate, cartilaginous rings of the trachea, intestinal villi, and feathers, scales, or hair follicles, develop in a periodically patterned manner. Although many specific models to explain the spontaneous emergence of such repeating patterns in embryonic tissues have been proposed [1], these can be grouped into 2 general classes (Fig 1A). The first class, based on the Turing reaction–diffusion system, relies on the operation of 2 opposing signalling processes: an activator, which is self-enhancing and has a limited spatial range, coupled with the production of an inhibitor with a greater spatial range. These activating and inhibiting processes were originally presented as a pair of chemical signals [2], though more complex networks can produce similar patterns [3–7]. These activating and inhibitory processes induce a spatially patterned change in cell state, typically reflected in altered gene expression. This arrangement of cell states, termed a prepattern, is then used as a template to produce anatomical structures by locally influencing cell aggregation, growth, or survival (Fig 1A). The second class of models focuses on the potential of mobile mesenchymal cells to generate periodic foci of high cell density directly through chemotaxis, adhesion, or mechanical deformation of their environment [8–13]. Such mesenchymal patterning does not require opposing intercellular signals, as the self-activation phenomenon arises from local cell clustering, while the inhibitory effect is achieved by widespread cell depletion as these are drawn away to nascent clusters (Fig 1A). Thus, these 2 theories differ in the entity that is moving: in the Turing reaction–diffusion system, cell movement is limited and chemical signals diffuse, while in mesenchymal self-organisation systems, moving cells are themselves agents of pattern formation. Both types of model produce similar patterns in computational simulation [4, 14–16] as they both ultimately rely on the principle of local self-activation and long-range inhibition [17]. Thus, experimental investigation is required to define the contribution made by each mechanism during organ development.

In the mouse embryo, a periodic pattern of hair follicles first arises at late embryonic day 13 (E13) [18, 19]. At this stage, the skin is composed of an epidermal sheet overlying an extracellular matrix containing dispersed mesenchymal cells. Sites of hair follicle initiation first become identifiable as groups of cells expressing specific marker genes and by cellular reorganisation (Fig 1B). The latter involves the closer packing of epidermal cells to form a placode [20] and the clustering of mesenchymal cells to form a dermal condensate directly underneath. Modulation of hair follicle size, shape, and spacing during skin growth and after experimental perturbation has provided the strongest evidence that a local self-activation and long-range inhibition system ultimately determines the hair follicle arrangement. Such a process can explain why new follicles are inserted between existing ones as the skin expands [21] and why the follicles will align along the edge of a cut made prior to pattern formation [4, 22]. This mechanism also explains how the hair follicle pattern can transition from an array of spots to one of stripes in a labyrinthine pattern [4]. As both cell–cell signalling and mesenchymal cell aggregation are prominent features of early hair follicle formation, it is plausible that either process, or a combination of both, is responsible for defining the hair follicle array.

Classical tissue recombination experiments indicated that the dermal mesenchyme is the compartment in which pattern generation occurs, this spatial information subsequently being conveyed to the epidermis through inductive signalling [23, 24]. This sparked a search for the molecular identity of the “first dermal message” thought to induce the epidermal placode pattern according to a dermal prepattern. Several intercellular signalling pathways have since been found to be critical for early hair follicle development, though none have the exact characteristics of the hypothesised first dermal message. Nascent follicle primordia are foci of high wingless-related integration site (WNT) activity [18, 25, 26] and display elevated production of fibroblast growth factors (FGFs) [27], bone morphogenetic proteins (BMPs), and BMP inhibitors [22, 28, 29]. Consistent with a driving role for the dermis in hair follicle patterning, this process does not initiate in mice upon abolition of dermal WNT/β-catenin activity [30]. However, activity of WNT/β-catenin in the epidermis is also essential for hair follicle patterning, as no focal expression of any molecular markers or cellular rearrangements that accompany normal hair follicle formation occur when this is ablated [25]. Furthermore, forced activation of epidermal β-catenin is sufficient to drive placode identity across the entire epidermis [31, 32]. Mutation of epithelial FGF receptor 2 leads to loss of cell rearrangements and placode markers [33], while mutation of *Fgf20* allows formation of epidermal placodes and expression of a nearly full suite of epidermal placode markers without any sign of an accompanying dermal condensate nor of patterned dermal gene expression [27]. However, administration of FGF7 to skin inhibits hair follicle formation [34], suggesting both positive and negative roles for FGF signalling in this process. The BMP family act as inhibitors of hair follicle formation, based on their effects when applied to cultured skin [21, 22, 29], an increased primary follicle density when epithelial BMP receptor is deleted [35], and the suppression of follicle formation when the BMP inhibitor NOGGIN is ablated [28].

Once the spatial pattern has been defined, the cells selected to become a follicle activate expression of other genes to progress their development into construction of the mature organ. These later-acting genes are typically expressed in the ‘de novo’ mode, indicating their activation only after assumption of the new cell fate, as opposed to the ‘restrictive’ mode of expression, which is characteristic of genes involved in the patterning process itself [36]. An example is *Shh*, which is expressed only once hair follicle cell fate has been established [37], and, agreeing with its role being only subsequent to definition of the follicle pattern, mutations in this gene do not impair hair follicle fate acquisition but rather arrest the follicles’ development due to lack of growth [38].

Simple signalling interactions have been proposed as the basis of hair follicle periodic patterning, though none represent a complete system capable of de novo pattern formation. WNT signalling coupled with induced expression of Dickkopf (DKK) family members was proposed to contribute to a reaction-diffusion system, though no WNT activator positive feedback loop was identified [39]. An activator/inhibitor relationship between ectodysplasin A receptor (EDAR) and the BMP pathway was suggested to contribute specifically to primary hair patterning [5, 22]. This system, however, stabilises a labile prepattern of focal WNT/β-catenin activity [22, 25, 40, 41], rather than acting to create order in naïve tissue. Thus, these proposed models do not integrate all pathways implicated in patterning and do not report a set of interactions sufficient to act as a periodicity generator, raising questions as to whether a simple reaction–diffusion system underlies hair follicle patterning or whether this process may have inputs other than intercellular signalling that contribute to symmetry breaking.

Here, we identify a set of interactions between the BMP, FGF, and WNT pathways capable of breaking symmetry to produce a prepattern that guides local dermal cell condensation. Strikingly, by modulating components of this system to mimic the microenvironment of the hair follicle primordium, we identify a transforming growth factor (TGF) β-driven patterning system in dermal mesenchyme that is independent of epidermal instruction. Whereas TGFβ signalling is capable of driving mesenchymal self-aggregation when reaction–diffusion signalling is suppressed, in normal development, this pathway potentiates cell accumulation at sites of focal FGF production, thereby assuring timely dermal condensate formation according to the epidermal template. Thus, both reaction–diffusion signalling and mesenchymal self-organisation potentials reside in the developing skin, but the former dominates and directs the latter, highlighting the hierarchical nature of patterning mechanisms.

## Results

### Regulatory relationships between BMP, FGF, and WNT in hair follicle patterning

To determine the functions and relationships between cell signalling pathways and whether their interactions are sufficient to constitute a complete pattern forming network, we focussed on the BMP, FGF, and WNT/β-catenin pathways [25–34]. We assessed the effects of modulating each pathway in E13.5 cultured skin on both epidermal placode, defined by expression of *Dkk4* [41, 42], and using the TCF/Lef::H2B-green fluorescent protein (GFP) reporter line [43] to visualise dermal cell arrangement and condensation (Fig 1C and S1C Fig). Treatment with BMP4 inhibited placode and dermal condensate formation, while the BMP receptor inhibitor LDN193189 increased placode and condensate size. We focused primarily on the FGF9/16/20 subfamily signalling as FGF family representatives due to their demonstrated involvement in hair follicle formation [27, 44]. Treatment with recombinant FGF9 suppressed placode and condensate appearance (Fig 1C), as did FGF7, a member of a different subfamily (S2 Fig). Blocking FGF signalling with SU5402 inhibited both *Dkk4* expression and dermal condensate appearance, consistent with FGF signalling being both necessary for, and inhibitory to, hair follicle development (Fig 1C). Treatment of skin explants with the GSK3β-inhibitor CHIR99021 to stimulate β-catenin activity led to the enlargement of hair follicle primordia, transitioning from discrete spots to a merged labyrinthine pattern. Inhibition of WNT/β–catenin signalling reduced placode density, accompanied by weaker expression of *Dkk4* (Fig 1C). In each condition, the effects on placode pattern were matched by corresponding changes in dermal condensate pattern. These pattern transitions are in agreement with WNT signalling being activatory, BMPs functioning as inhibitors, and FGFs playing a more complex role, being both required for and inhibitory to hair follicle initiation.

Having defined conditions to stimulate and repress each of these 3 signalling pathways, we set out to delineate their transcriptional regulatory interactions and assess whether these could comprise a functional pattern forming network. Turnover of activator and inhibitor molecules is required to produce a reaction–diffusion pattern [2, 4, 17], and simulations show that activator and inhibitor half-lives have a major influence on the timing of pattern emergence [4]. As mouse skin takes approximately 10 h to form a pattern from a naïve state [22], in a simple reaction–diffusion network, this constrains the half-lives of the oscillating components of this system to being under 90 minutes [4]. We used this temporal constraint as a filter to identify transcripts from these 3 pathways that could play a driving role in pattern formation.

We performed a transcriptome-wide screen of mRNA half-lives in E13.5 mouse dermis and epidermis, subsequently applying a data filtering criterion based on half-life (t_1/2_ ≤ 90 minutes) and focusing upon extracellular molecules (ligands, receptors, and extracellular signal-attenuating molecules) of the BMP, FGF, and WNT pathways (S3 Fig, S2 and S3 Tables, S1A Appendix for detail). The focus on ligand, receptor, and extracellular antagonist molecules is motivated by their critical role in reaction–diffusion processes by conveying information between cells [2, 17, 45]. This yielded a candidate list of mRNA species that fit within the 3 signalling pathways of interest, have half-lives of less than 90 minutes, encode extracellular proteins, and are expressed in embryonic skin (S1 Table). This set contains many genes already implicated in skin patterning, including *Bmp2* and *Bmp4* [21, 22, 29], the BMP inhibitor *Nog* [28, 46], the WNT inhibitors *Dkk1* and *Dkk4* [26, 39, 41, 47], and *Fgf7* and *Fgf20* [27, 34]. Transcripts encoding proteins in the EDAR and TGFβ pathways had longer half-lives, consistent with their acting later to stabilise emerging patterns [22] and promote morphogenesis [48].

To identify regulatory relationships between the BMP, FGF, and WNT pathways, we stimulated and repressed each pathway in unpatterned E13.5 skin explants for 6 h and assessed the resulting change in abundance of each candidate transcript (Fig 1D). This approach identified a number of previously reported regulatory interactions, including upregulation of *Fgf20* and *Dkk4* by WNT activity [27, 41, 42] and stimulation of *Bambi* and *Nog* by BMP signalling [49, 50].

After excluding candidates unresponsive to BMP, FGF, or WNT signal modulation, 15 genes were found to be regulated within this period (*Bmp2*, *Bmp4*, *Ctgf*, *Dkk1*, *Dkk4*, *Wnt9a*, *Ror2*, *Fgf7*, *Fgf10*, *Fgf18*, *Fgf20*, *Rgma*, *Fzd10*, *Bambi*, and *Nog*). From these data, we derived a transcriptional network describing the interactions between the candidates (Fig 1E). In this network, WNT/β–catenin signalling stimulates expression of patterning genes and hair follicle primordium markers, while BMP has opposing effects on most of these. FGF represses expression of all epithelial WNT/β–catenin activated targets (*Dkk4*, *Bmp2*, and *Fgf20*), which are indicators of placode fate, but does not alter expression of any dermally expressed genes in the network, even though we detect the classical FGF target *Etv5* being upregulated in both tissue layers (S4A Fig). The inhibition of hair follicle development following widespread application of FGF to skin cultures (Fig 1C) can thus be explained by its suppression of epithelial β-catenin activity, consistent with the increased epidermal β-catenin activity reported in *Fgf20* null embryonic skin [27] and reduction of active β-catenin protein in FGF-stimulated epidermis (S4B and S4C Fig).

In this network, some features of classical Turing activator–inhibitor dynamics are present, including activator stimulation of inhibitor production, but we did not detect direct positive feedback for any pathway. Rather, each pathway displays prominent self-inhibition. Thus, in its number of components and its structure, this network does not conform to the classical topology of a reaction–diffusion system. Therefore, we took a mathematical approach (S1B Appendix) to assess whether the structure of this network is capable of producing a periodic pattern.

We grouped the candidates into 5 species: BMP (*Bmp2*, *Bmp4*, *Rgma*), WNT (*Wnt9a*, *Ror2*, *Fzd10*), WNT inhibitor (*Dkk1* and *Dkk4*), BMP inhibitor (*Bambi* and *Nog*), and FGF (*Fgf20*) (see S1A Appendix). We then defined a matrix in which the interactions between these species were represented by either a + sign (stimulation) or a − sign (inhibition) (S3F Fig). From the matrix of interactions, we performed linear stability analysis [2], a method that assesses whether small perturbations in a system will decrease or increase over time. If they decay, patterning cannot occur, and if they grow, there is a prospect of pattern formation, as a result of diffusion in the case of the Turing mechanism. This analysis found that the conditions of Turing instability are met when all 5 species are considered to be diffusible (Fig 1F), which is expected as each grouping contains at least 1 secreted factor. The structure of this BMP, FGF, and WNT network can, therefore, generate a stable periodic pattern (see S1C Appendix for detail) through rapid regulatory interactions. Intuitively, the main drivers in this system lie in WNT stimulating expression of a suite of hair follicle primordium-specific genes, while BMP broadly inhibits their expression and FGF acts as an inhibitor selectively in the epidermis. While direct positive feedback is not apparent in this system, the sequence through which WNT upregulates FGF, FGF downregulates BMP, and BMP downregulates WNT could have an overall positive effect that would allow WNT to undergo indirect self-upregulation.

### Dermal condensates are generated by directed local mesenchymal cell movement

**Fig 2:**
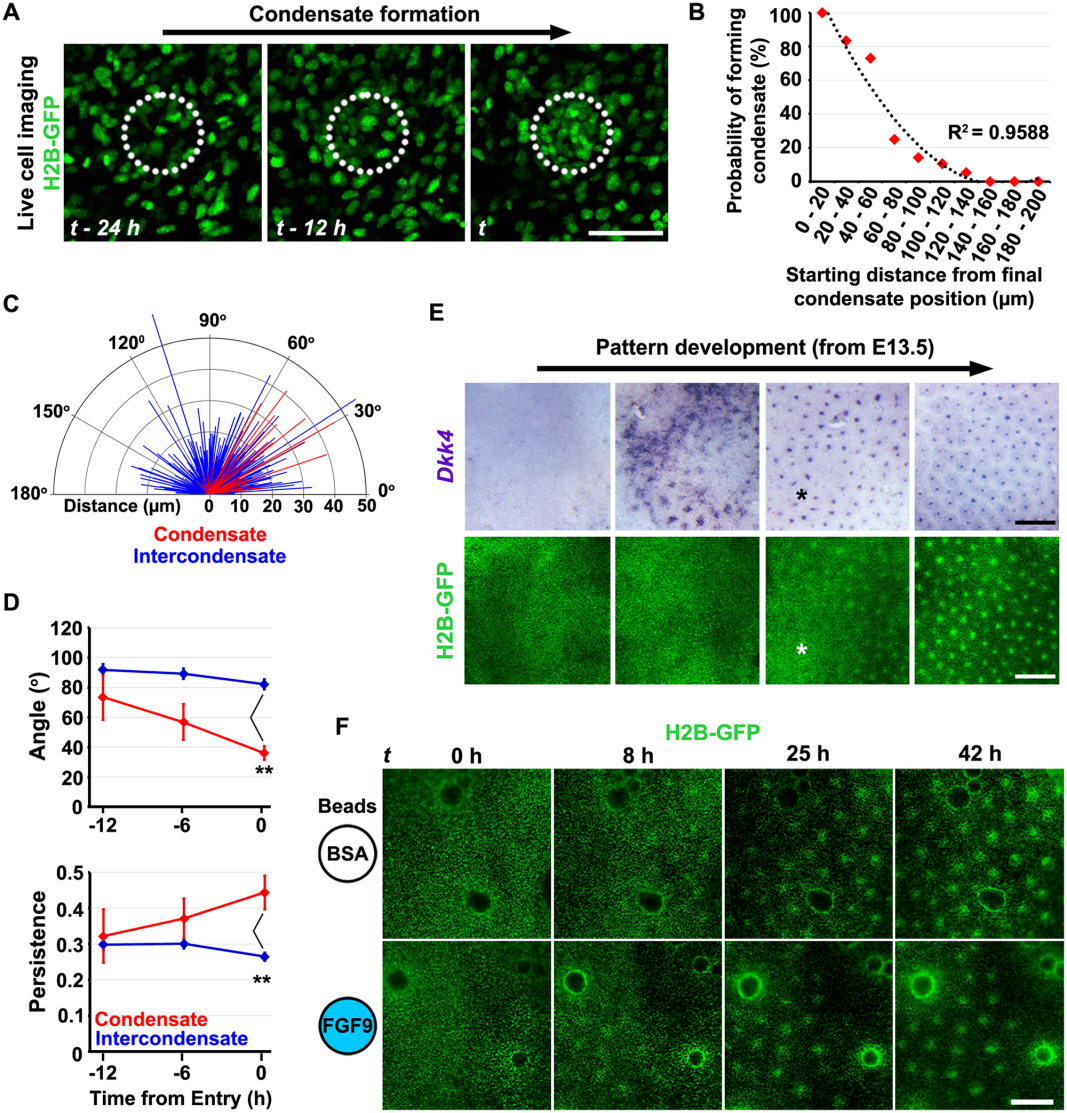
Dermal condensate formation occurs after epidermal patterning through local cell attraction. (A) Single frames from time-lapse sequences of E13.5 TCF/Lef::H2B-green fluorescent protein (GFP) skin explant culture captured by confocal microscopy. Dashed circles indicate ultimate condensate location. Scale bar: 50 μm. (B) Analysis of tracked cells showing the probability of joining the dermal condensate based upon initial location relative to its centre. Two hundred and forty individual cells were tracked across 8 condensates from 4 independent skins. (C) Protractor plot showing the distribution of Euclidean angles and Euclidean distances of individual cell movements in 6-h windows for cell tracks that start outside of, but ultimately terminate in, a follicle (condensate = red) and those that remain outside (intercondensate = blue). Tracking was halted on cell entry. (D) Plots showing the mean Euclidean angle (top) and mean level of persistence (bottom) of condensate-entering and intercondensate cells for 6-h windows relative to time of entry into the condensate. Error bars represent SEM (condensate cells *n* = 9, 14, and 20 and intercondensate *n* = 263, 245, and 197 for 12, 6, and 0 h before entry, respectively). Statistical significance was calculated using a Kruskal–Wallis test (*p* < 0.0001 and *p* < 0.001 for angle and persistence, respectively) followed by Mann–Whitney U tests with Bonferroni’s correction (**p < 0.01). The raw numerical tracking data (for B, C, and D) can be found in S2 Data. (E) Detection of a molecular prepattern prior to dermal condensate formation. TCF/Lef::H2B-GFP skin explants were fixed at intermediate stages of pattern formation, imaged to detect GFP, and *Dkk4* expression determined in the same skin sample. Asterisk represents an area where *Dkk4*-positive foci are present but corresponding dermal condensates are absent. Scale bar: 500 μm. (F) Time-lapse images of E12.75 TCF/Lef::H2B-GFP dorsal skin explants cultured with recombinant fibroblast growth factor (FGF) 9- or bovine serum albumin (BSA)-loaded beads. Cells accumulate around FGF9-loaded beads. Scale bar: 250 μm.

Having defined the reciprocal relationships between BMP, FGF, and WNT signalling, including their organisation into a network capable of periodic patterning, we investigated the origin of the dermal condensate and the influences of these pathways on its formation. We performed live cell imaging of TCF/Lef::H2B-GFP E13.5 dorsal skin explants using confocal microscopy (Fig 2A, S1 and S2 Videos) to track dermal cells during condensate formation. This revealed undirected movement of dermal cells up to the timepoint of condensate formation, when local directed cell movement produces the structure. Condensate-entering cells were observed to do so individually, with no sign of collective migration (S1 Video). We found that the dermal cells which ultimately make up the condensate are those present in its immediate vicinity, with a simple relationship between initial cell location and probability of incorporation into the condensate (Fig 2B).

We performed an analysis of the direction of movement of individually tracked cells by dividing each cell track into 6-h windows and determining the Euclidean angle of movement with respect to the future condensate centre for each window, as well as the Euclidean and accumulated distance travelled over this period. To determine whether individual cell movement was directed, we compared the distribution of Euclidean angles and distances for 2 classes of cells: (i) those cells initially outside the condensate area which subsequently entered it (‘condensate’) and (ii) those cells which remained outside the condensate area throughout (‘intercondensate’) (Fig 2C). Intercondensate Euclidean angles (*n* = 893) in these windows did not deviate from a uniform distribution (Kolmogorov–Smirnov test *D* = 0.05, *p* > 0.05) while condensate-bound track angles (*n* = 45) were consistently lower (Kolmogorov–Smirnov test *D* = 0.44, *p* < 0.001). The median Euclidean distance travelled in these 6-h windows was also greater for condensate-bound cells (Mann–Whitney U test *p* < 0.0001) than intercondensate cells (Fig 2C). Condensate-entering cells therefore exhibit directed movement towards sites of follicle initiation.

To investigate the timing of the distinct behaviours of cells entering condensates, we quantified cell behaviour by comparing the three 6-h windows prior to follicle entry (or the end of the track for intercondensate cells). We compared the Euclidean angle and level of persistence (calculated as the Euclidean distance/accumulated distance) of movement by the 2 cell classes (intercondensate and condensate) at 0–6, 6–12, and 12–18 h prior to follicle entry (Fig 2D). The median Euclidean angle relative to the nearest prospective condensate centre differed significantly between intercondensate and condensate-bound cells in the 0–6 h window (Kruskal–Wallis test *p* < 0.0001, Mann–Whitney U test with Bonferroni’s correction *p* < 0.01). Consistent with this behaviour, the median persistence for condensate-bound cells also differed from intercondensate cells in the 0–6 h window (Kruskal–Wallis test *p* < 0.01, Mann–Whitney U test with Bonferroni’s correction *p* < 0.01). These results show that the directed cell movement in the condensate-entering cell population occurs locally and only in the hours immediately prior to condensate appearance. Prior to this time, the behaviour of the entire mesenchymal cell population is the same, indicating that condensate formation involves selection of cells from an equivalent population based simply on their location.

### Dermal cell condensation is directed by an epidermal prepattern

Using the TCF/Lef::H2B-GFP line, we set out to determine the relative order of placode specification and cell condensation. We fixed cultured dorsal skin cultures at intermediate stages of development and compared the timing of appearance of *Dkk4* expression with that of cell condensates in individual samples of skin. We found that patterning is first detectable in the epidermis as spatially organised focal *Dkk4* expression with a lack of corresponding dermal cell clustering (Fig 2E). As patterning continues, dermal condensates become apparent, and the foci of *Dkk4* expression resolve. However, at this stage, not all epidermal placodes have corresponding dermal condensates, and there are regions of skin where the epidermal pattern is present in the absence of dermal organisation. As the process completes, each *Dkk4*-positive placode is underlain by a dermal condensate (Fig 2E). These results show that an epidermal gene expression prepattern precedes the formation of dermal condensates.

Based on these findings, we hypothesised that local signalling from the epithelial placode might coordinate the condensation of the underlying dermal cells. We considered FGF a good candidate for the local attractant signal as this induces dermal condensations during feather development [51, 52] and because knockout mice lacking *Fgf20*, a gene selectively expressed in epidermal placodes, do not form dermal condensates [27]. To investigate whether a local FGF source could attract dermal cells, we cultured E12.75 TCF/Lef::H2B-GFP dorsal skin explants with beads soaked in recombinant FGF9 or control protein bovine serum albumin (BSA) beads. FGF9 beads induce cell accumulation in their vicinity, unlike BSA beads (Fig 2F and S3 Video). The area surrounding the FGF9 bead-induced condensate contains fewer GFP^+ve^ nuclei, indicative of cell depletion from this area, and exhibits lack of hair follicle primordia (Fig 2F). Together, these observations show that local sources of FGF stimulate dermal condensate formation, demonstrating a positive role for FGF in hair follicle formation in the mesenchyme, in contrast to its effect on the epidermis.

### BMP destabilises dermal condensates

**Fig 3:**
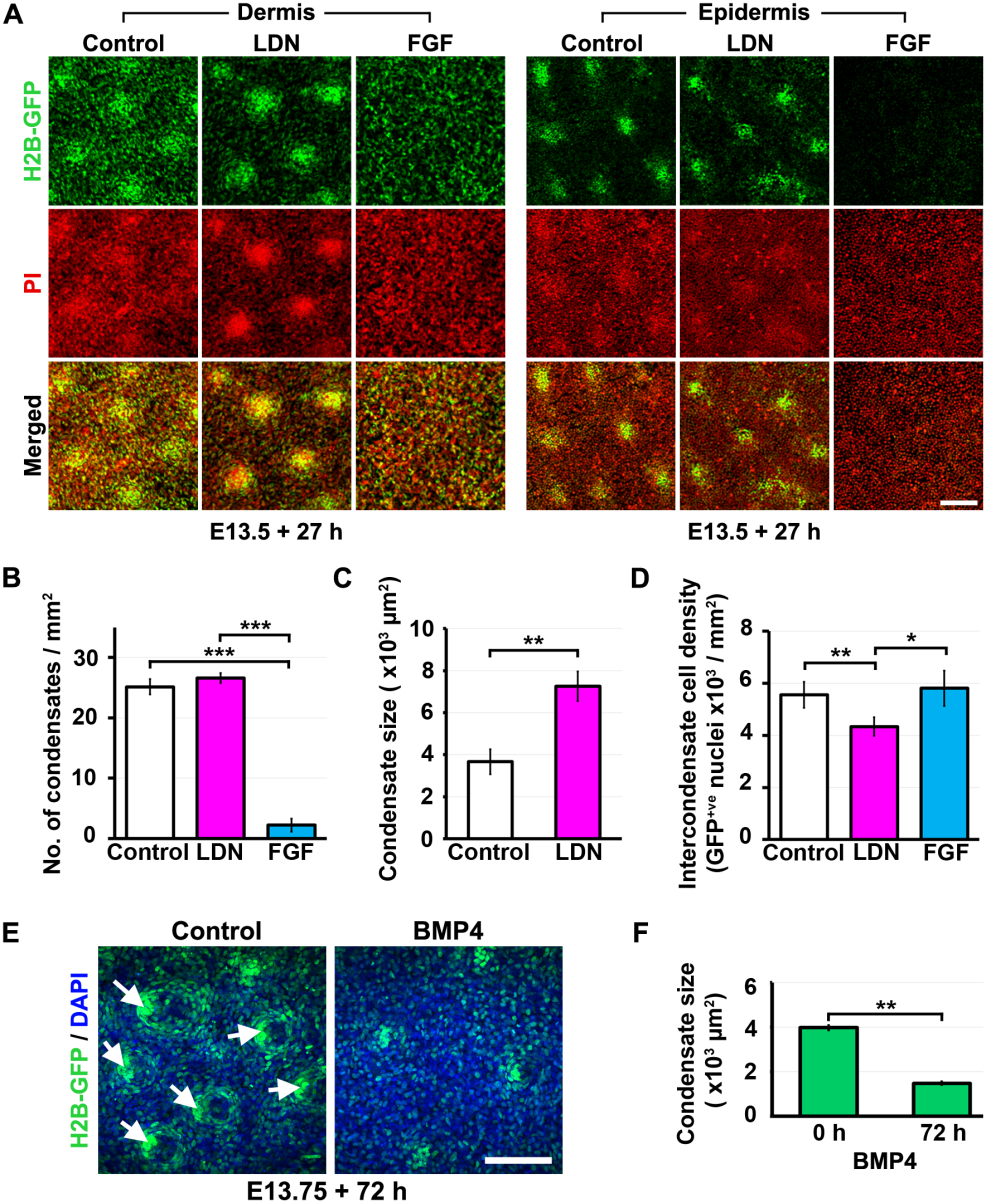
BMP destabilises mesenchymal aggregates. (A) Epidermis and dermis isolated from E13.5 TCF/Lef::H2B-green fluorescent protein (GFP) skin explants cultured with LDN193189 (LDN) (10 μM) or fibroblast growth factor (FGF) 9 (1 μg/ml) for 27 h, counterstained with propidium iodide (PI) and imaged using confocal microscopy. Scale bar: 100 μm. (B) Number of condensates per square mm, (C) mean condensate area, and (D) intercondensate cell density measured in E13.5 TCF/Lef::H2B-GFP skin explants cultured with either LDN or FGF. Error bars represent SEM from 5 independent experiments. Significant difference was calculated using a Student paired *t* test (*p < 0.05, **p < 0.01, ***p < 0.001). (E) E13.75 TCF/Lef::H2B-GFP skin explants (with pre-existing dermal condensates) were treated with bone morphogenetic protein (BMP) 4 for 72 h. Skins were counterstained with 4’6-Diamidino-2-phenylindole dihydrochloride (DAPI) and confocal imaged for GFP at 72 h. Scale bars: 100 μm. (F) Quantification of individual condensate area following BMP supplementation. Error bars represent SEM from at least 3 independent experiments. Significant difference was tested using a paired Student *t* test (*p < 0.05, **p < 0.01, ***p < 0.001). The raw numerical values (for B, C, D, and F) can be found in S3 Data.

Having established that FGF attracts mesenchymal cells to form a condensate and knowing that reception of WNT signals by mesenchymal cells is also required for this process [30, 53], we set out to define the effects of BMP signalling on dermal cell organisation. TCF/Lef::H2B-GFP skin explants treated with the BMP inhibitor LDN193189 have enlarged dermal condensates and modestly increased placode size (Fig 3A and 3C). We found that inhibition of BMP signalling significantly reduced the density of nuclei in the area between condensates (Fig 3A and 3D), indicating that increased dermal condensate size results from greater recruitment of dermal cells to produce enlarged follicle primordia. Thus, active BMP signalling normally limits cell recruitment to incipient condensates, and when BMP is impaired condensates continue to expand through cell recruitment, depleting cells from the intercondensate region.

To further define the influence of BMP on dermal cell arrangement, we stimulated this pathway in skin cultures containing existing dermal condensates. BMP treatment led to erosion of condensates, with a profound reduction in their size (Fig 3E and 3F). This demonstrates that BMP stimulation destabilises and depletes cells from mesenchymal aggregates when present in excess. Although dermal condensates are sites of high BMP4 production, BMP activity within the hair follicle rudiments is normally restrained through expression of the inhibitors NOGGIN and CTGF in the condensate and placode, respectively [22, 28, 29]. This, together with selective *Fgf20* expression in the placode [27], produces a niche microenvironment of low BMP and high FGF activity at sites of follicle formation.

### Tuning of BMP and FGF signalling permits mesenchymal self-organisation without epidermal patterning

**Fig 4:**
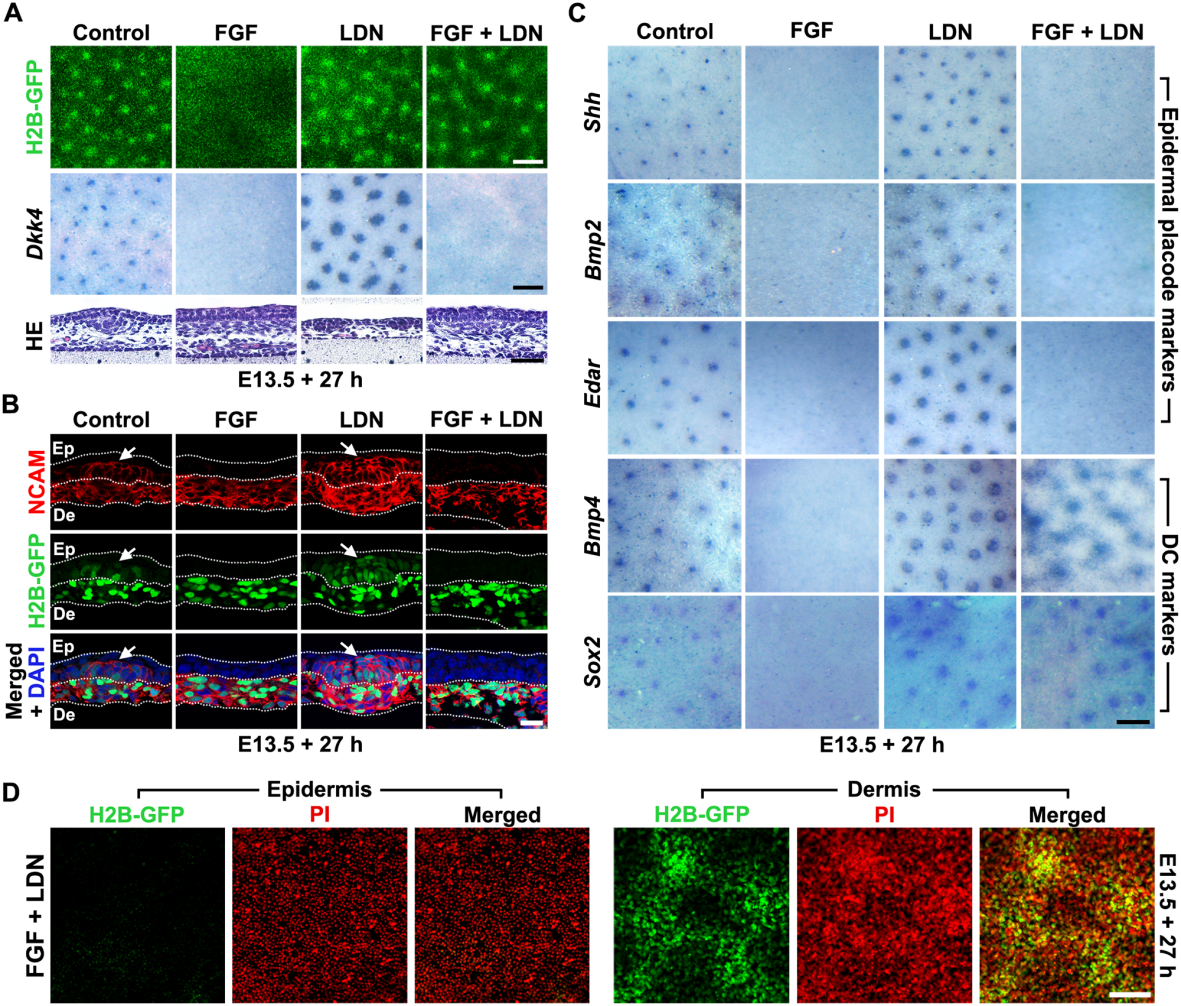
Spatially patterned dermal condensates can form in the absence of epidermal placodes. (A) Detection of green fluorescent protein (GFP) signal, *Dkk4* expression, and histological appearance of TCF/Lef::H2B-GFP skin explants cultured with fibroblast growth factor (FGF) 9 (1 μg/ml), LDN193189 (LDN) (10 μM), or both agents. Large, periodically spaced dermal condensates form in the absence of epidermal placodes when FGF and LDN are administered. Scale bars: H2B-GFP & *Dkk4*: 250 μm, haemotoxylin and eosin (HE): 50 μm. (B) Frozen sections of TCF/Lef::H2B-GFP dorsal skin explants treated as indicated and stained for neural cell adhesion molecule (NCAM) expression. Arrows indicate epidermal placode. Scale bar: 20 μm. (C) Expression of epidermal placode (*Shh*, *Edar*, *Bmp2*) and dermal condensate (*Bmp4*, *Sox2*) marker genes in dorsal skin explants cultured with FGF and LDN. Scale bar: 250 μm. (D) Epidermis and dermis isolated from E13.5 TCF/Lef::H2B-GFP skin explants cultured with FGF9 and LDN, counterstained with propidium iodide (PI) and imaged using confocal microscopy. Epidermis is unpatterned while the large dermal condensates are accompanied by cell depletion from the intervening spaces. Scale bar: 100 μm.

These results indicate roles for FGF and BMP signalling in influencing mesenchymal cell aggregation. We tested whether these influences could be modulated to trigger mesenchymal condensation by mimicking the hair follicle primordium microenvironment—that is, high-FGF and low-BMP signalling (hereafter, FGF^Hi^BMP^Lo^)—across the entire skin. Strikingly, imposing these conditions using FGF9 together with LDN193189 caused dermal condensates to arise in a periodically spaced manner without corresponding expression of the epidermal placode marker *Dkk4* (Fig 4A). Histological sections from these samples showed the presence of large dermal condensates at the dermal–epidermal junction but an absence of epidermal placodes (Fig 4A). Neural cell adhesion molecule (NCAM) staining reveals the continued dermal identity of cells in the condensates thus induced, together with the absence of the distinct placodal NCAM expression that indicates an epidermal contribution to patterning (Fig 4B). As observed for *Dkk4*, other markers of epidermal placodes (*Shh*, *Bmp2* and *Edar*) [18, 54] are not expressed in FGF9- and LDN193189-treated skins, while dermal condensate markers *Bmp4* and *Sox2* [54, 55] do display patterned expression, demonstrating the dermal condensate identity of the mesenchymal aggregates (Fig 4C). In FGF^Hi^BMP^Lo^ conditions, epidermal TCF/Lef::H2B-GFP reporter signal is not detectable, demonstrating that FGF suppression of epidermal β-catenin signalling is not alleviated by simultaneous inhibition of BMP signalling. The dermal condensates that form under FGF^Hi^BMP^Lo^ conditions have increased overall condensate size and markedly low cell density between the broad condensates (Fig 4D). The ability to pattern mesenchymal condensates in an FGF^Hi^BMP^Lo^ environment is not restricted to a specific developmental stage (S5 Fig), and these structures, once induced to form, are autonomously stable upon restoration of normal FGF and BMP function (S6 Fig).

To determine the relative rates of dermal condensate patterning, we cultured skin explants under normal or FGF^Hi^BMP^Lo^ conditions and imaged the samples at different time points. Pattern formation under normal conditions is more rapid than in FGF^Hi^BMP^Lo^ conditions, with the control pattern appearing and stabilising quickly, while the FGF^Hi^BMP^Lo^ pattern is slower to appear (S7 Fig).

### Equivalent cell behaviours underlie recruitment-driven and mesenchyme-autonomous dermal condensate formation

**Fig 5:**
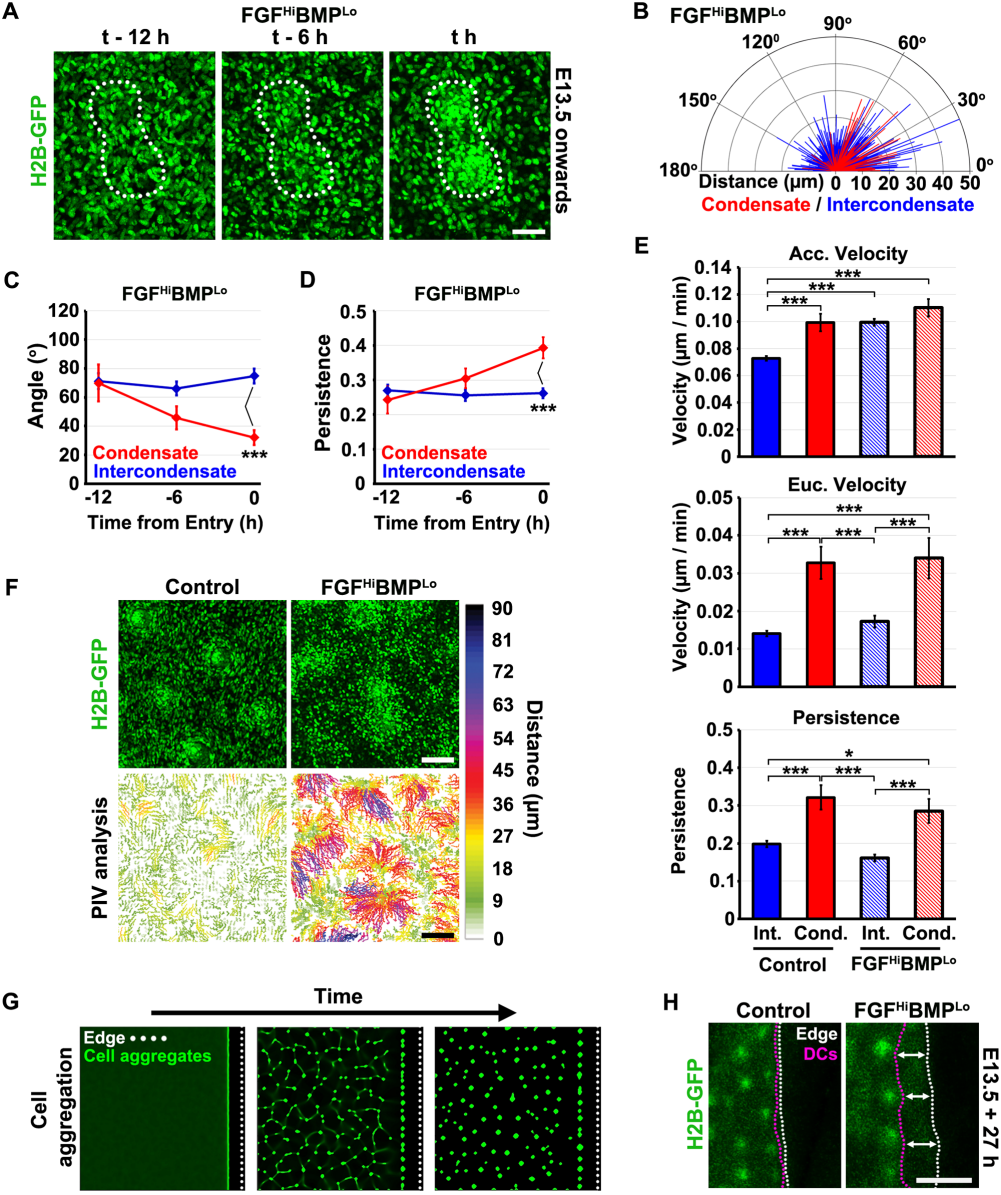
Cell behaviours underlying mesenchymal self-organisation. (A) Time-lapse images showing dermal condensate formation in FGF^Hi^BMP^Lo^ conditions. Scale bar: 50 μm. (B) Protractor plot showing the distribution of Euclidean angles and Euclidean distances of individual cell movements in 6-h windows for cell tracks that start outside of, but ultimately terminate in, a follicle (condensate = red) and those that remain outside (intercondensate = blue) under FGF^Hi^BMP^Lo^ conditions. Tracking was halted on cell entry. Plots showing (C) the mean Euclidean angle and (D) the mean level of persistence of condensate and intercondensate cells for 360-minute windows relative to time of entry into the condensate. From 6 h before entry, the condensate-bound cells show oriented and persistent movement under FGF^Hi^BMP^Lo^ conditions. Error bars represent SEM (condensate cells *n* = 17, 21, and 25 and intercondensate *n* = 91, 104, and 108 for 12, 6, and 0 h before entry, respectively). Statistical significance was calculated using a Kruskal–Wallis test followed by Mann–Whitney U tests with Bonferroni’s correction (***p < 0.001). (E) Comparison between per track summaries of condensate (Cond.) and intercondensate (Int.) cells under control or FGF^Hi^BMP^Lo^ conditions for (top) accumulated velocity, (middle) Euclidian velocity, and (bottom) persistence. Statistical significance was calculated using a Kruskal–Wallis test followed by Mann–Whitney U tests with Bonferroni’s correction (*p < 0.05, ***p < 0.001). Error bars represent SEM (control intercondensate *n* = 292, control condensate *n* = 28, FGF^Hi^BMP^Lo^ intercondensate *n* = 137 and FGF^Hi^BMP^Lo^ condensate *n* = 33). Raw tracking data for (B–E) can be found in S2 Data. (F) Particle image velocimetry analysis of normal and FGF^Hi^BMP^Lo^ condensate formation over 30 h. Coloured tracks show very local cell movement in control conditions but a much broader field of recruitment for the mesenchyme-only patterned condensates. Colour scale shows track length. Scale bar: 100 μm. (G) Simulation of boundary effects on patterning in chemotactic aggregation-driven patterning. (H) Experimental test of pattern behaviours. Distinct pattern behaviours at tissue edges. Under control conditions, primordia align along the edge. FGF^Hi^BMP^Lo^ condensates align with but form at a distance from boundaries introduced in skin explants prior to pattern formation. White dotted lines indicate the boundary. Magenta dotted lines indicate the extent of the patterned region where dermal condensates form. Scale bar: 250 μm.

Time-lapse imaging of the formation of mesenchymal condensates under FGF^Hi^BMP^Lo^ conditions reveals the gradual emergence of these focal aggregates across the entire skin (Fig 5A, S4 and S5 Videos). To investigate the cell behaviours underlying mesenchymal self-organisation, we performed live cell imaging of TCF/Lef::H2B-GFP E13.5 dorsal skin explants using confocal microscopy (Fig 5B–5D). We used the 6-h window analysis as before to determine the Euclidean angle of movement with respect to the future condensate centre for each window, as well as the Euclidean and accumulated distance travelled over this period. In FGF^Hi^BMP^Lo^ conditions, the intercondensate Euclidean angles (*n* = 612) deviate from a uniform distribution (Kolmogorov–Smirnov test *D* = 0.09, *p* < 0.01), while condensate-bound track angles (*n* = 91) were consistently altered from both a uniform distribution (Kolmogorov–Smirnov test *D* = 0.32, *p* < 0.001) and the distribution of intercondensate angles (Kolmogorov–Smirnov test *D* = 0.25, *p* < 0.001). As observed under normal (control) conditions (Fig 2C), the median Euclidean distance travelled in these 6-h windows was greater for condensate-bound cells (Mann–Whitney U test *p* < 0.001) than intercondensate cells (Fig 5B).

The behaviour of these cells entering condensates is remarkably similar to that observed in conditions of normal patterning (Fig 2C and 2D), with cells showing directed movement to condensates (Fig 5B–5D). The median Euclidean angle of movement differed significantly between intercondensate and condensate-bound cells in the 0–6-h window (Kruskal–Wallis test *p* < 0.001, Mann–Whitney U test with Bonferroni’s correction test *p* < 0.001) (Fig 5C). Consistent with this behaviour, the median persistence for condensate-bound cells also differed from intercondensate cells in the 0–6-h window (Kruskal–Wallis test *p* < 0.001, Mann–Whitney U test with Bonferroni’s correction *p* < 0.001) (Fig 5D), further demonstrating directed movement of condensate-entering cells.

To further investigate cell behaviour between normal (control) and FGF^Hi^BMP^Lo^ conditions, we compared summary statistics for individual cell tracks (Fig 5E). As expected, the median accumulated velocity of condensate-entering cells in normal and FGF^Hi^BMP^Lo^ conditions (Fig 5E) was increased when compared to intercondensate cells under control conditions (Kruskal–Wallis test *p <* 0.0001, Mann–Whitney U test with Bonferroni’s correction *p* < 0.001 and *p* < 0.0001, respectively). However, surprisingly, the median accumulated velocity of intercondensate cells in the FGF^Hi^BMP^Lo^ conditions was also significantly higher than intercondensate cells in control conditions (*p* < 0.0001) (Fig 5E). This was not reflected by a change in the median Euclidean velocity nor the persistence of intercondensate cells under FGF^Hi^BMP^Lo^ conditions (Kruskal–Wallis test *p <* 0.0001, Mann–Whitney U test with Bonferroni’s correction *p* > 0.05 for both cases), suggesting that the increase in cell movement was due to a chemokinetic effect of FGF^Hi^BMP^Lo^ conditions (Fig 5E). Taken together, these results show that slower pattern emergence under FGF^Hi^BMP^Lo^ conditions is not a result of sluggish cell movement but a reflection of the dynamics of the pattern-forming process itself. To gain an overall view of cell displacement across the field during the course of cellular condensation, we used particle image velocimetry (see S1D Appendix) to analyse the time-lapse videos (S1 and S5 Videos). This approach delineates the average paths of cells (Fig 5F, see S1D Appendix) revealing that a major distinction between unperturbed and mesenchyme-only patterning is the large zone of attraction which extends to collect cells in the latter condition.

As the underlying individual cell behaviour driving condensate formation is the same in control and FGF^Hi^BMP^Lo^ conditions, we asked whether these are fundamentally distinct patterning mechanisms or whether they might be differently regulated outputs of a single underlying mechanism. Reaction–diffusion- and cell aggregation-based patterning mechanisms behave differently at tissue boundaries [4, 22, 56, 57]. Simulations of reaction–diffusion systems display an edge affinity in the arrangement of their cell clusters [4]. In contrast, simulations of cell aggregation-based systems display cell clusters that form at a distance from the cut edge (Fig 5G, see S1E Appendix). We manipulated the tissue boundaries under both control and FGF^Hi^BMP^Lo^ conditions by introducing a cut edge into the skin explants and found, consistent with the mesenchyme-only patterning arising from a different mechanism, edge effects to be different for normal versus mesenchyme-only patterning. The normal pattern aligns close to the cut edge of the tissue, as previously reported [4, 22], while the latter respects the shape of the boundary but maintains a large distance from the edge to the nearest pattern foci (Fig 5H). Intuitively, these behaviours can be thought of as recognising an advantage for cells adjacent to an edge in a reaction–diffusion system, as they lack competition on 1 side and are able to dilute the inhibitor that they produce into the culture medium. Conversely, in a patterning system based on cell aggregation, the cells close to the edge are disadvantaged, as they have reduced numbers of neighbours with which to nucleate clustering.

### Locally restricted TGFβ signalling drives mesenchymal self-organisation

As the mesenchymal patterning mechanism is fundamentally distinct from the normal condition, we sought a mechanistic basis for this process. We treated skin cultures with modulators of pathways previously implicated in dermal cell condensation to identify signalling pathways with selective effects on mesenchyme-only patterning. We found that mesenchyme, in both control and FGF^Hi^BMP^Lo^ conditions, patterned robustly, despite the presence of inhibitors or activators of the CXCL/CXCR, Notch, or platelet-derived growth factor (PDGF) pathways (S8 Fig).

**Fig 6:**
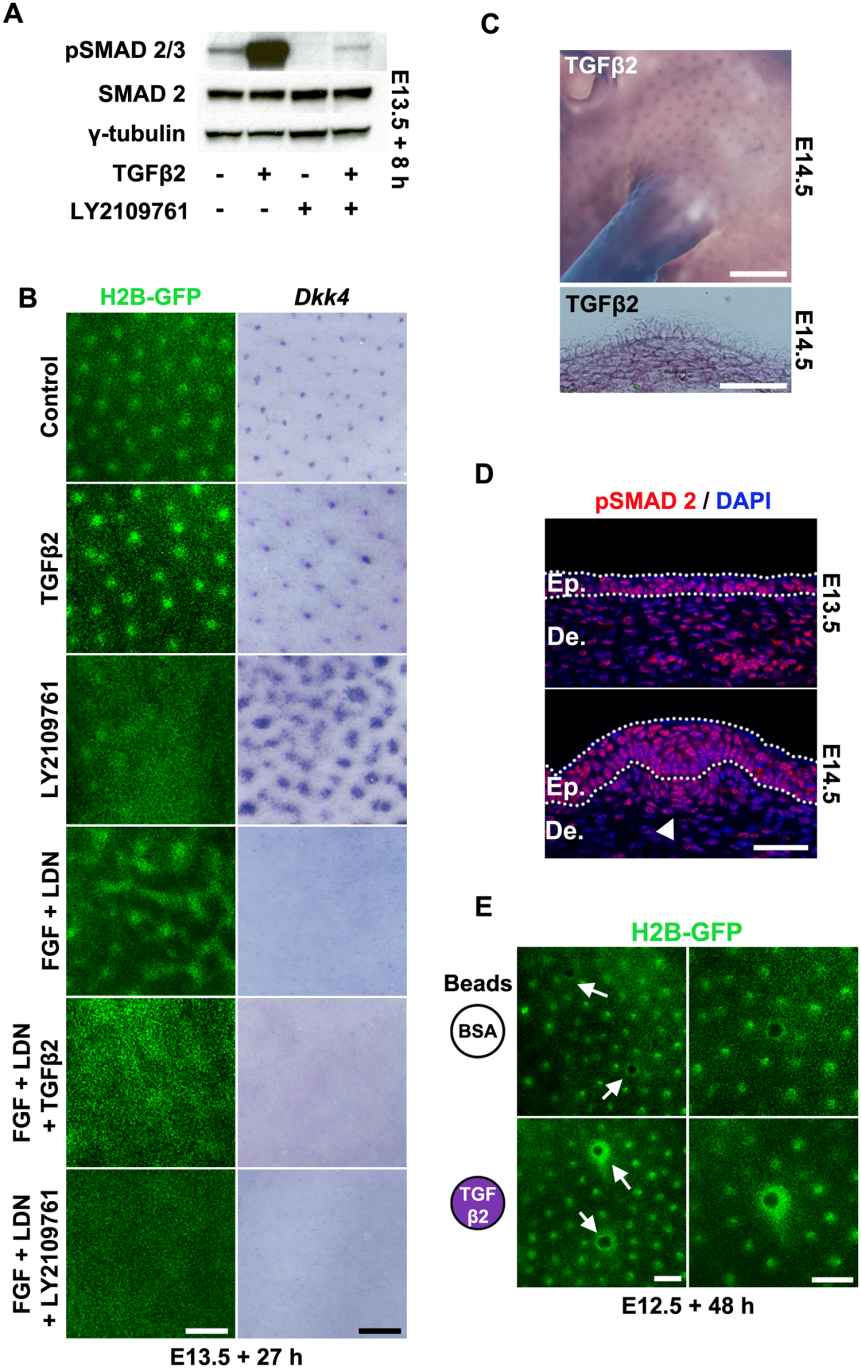
Dependence of mesenchyme-only patterning on restricted TGFβ availability. (A) Western blot detection of phospho-SMAD2, total SMAD2 and γ-tubulin in E13.5 skin cultures treated with recombinant transforming growth factor (TGF) β2 (100 ng/ml), the TGFβ receptor inhibitor LY2109761 (25 μM,) or both agents for 8 h. (B) Effects of TGFβ2 supplementation and LY2109761 on normal and mesenchyme-only patterning. Condensates are slow to appear in LY2109761, and expression of the placode marker *Dkk4* expands through the epidermis. Mesenchyme-only patterning in FGF^Hi^BMP^Lo^ conditions is abolished upon either suppression or augmentation of TGFβ signalling. Scale bars: 250 μm. (C) Whole-mount in situ hybridisation (top panel) and corresponding transverse section (bottom panel) detecting spatial arrangement of *Tgfb2* expression in E14.5 mouse embryos. Expression is most intense at sites of dermal condensate formation. Scale bars: top panel = 1 mm, bottom panel = 50 μm. (D) At E13.5, phospho-SMAD2 immunofluorescence detects signal throughout the dermal mesenchyme (De.) and epidermis (Ep.), with this signal becoming intensified in the nascent dermal condensate at E14.5 (arrowhead). Epidermis is demarcated by dotted lines. Scale bar: 25 μm. (E) Dermal mesenchymal cell attraction (arrows) to sources of TGFβ2. Images of bovine serum albumin (BSA) control and TGFβ2 loaded beads placed on E12.5 TCF/Lef::H2B-green fluorescent protein (GFP) skin for 48 h. Scale bars: 250 μm.

Alteration of TGFβ signalling, however, profoundly suppressed mesenchyme-only patterning while having relatively modest effects on normal patterning. The TGFβ receptor type I and II inhibitor LY2109761 (Fig 6A) slowed the assembly of normal dermal condensates, while placode patterns became expanded and threaded through the epidermis (Fig 6B and S9 Fig), matching the delayed hair follicle phenotype reported for the *Tgfb2* mutant mouse [48]. Augmenting signalling by administration of recombinant TGFβ2 permitted a normal array of placodes and condensates to arise, with condensates appearing more prominent than in control conditions. However, either suppression or widespread stimulation of TGFβ2 signalling abolished the ability of mesenchyme to pattern autonomously in FGF^Hi^BMP^Lo^ conditions (Fig 6B). Thus, active TGFβ signalling is required for mesenchyme-only patterning but must be restricted for this process to yield a spatially organised array of condensates.

*Tgfb2* is broadly expressed by mesenchymal cells [54, 58] during primary hair follicle formation and in cell clusters at sites of dermal condensation (Fig 6C). This expression is consistent with widespread SMAD2 phosphorylation in early dermal fibroblasts, becoming focussed in incipient dermal condensates (Fig 6D) [59]. To test whether TGFβ2 can attract mouse dermal mesenchymal cells, as previously reported for chicken mesenchyme [60], we applied TGFβ2-coated beads to skin and detected a strong accumulation of cells around the bead (Fig 6E). Thus, TGFβ2 represents a widely expressed attractant serving to draw mesenchymal cells together.

**Fig 7:**
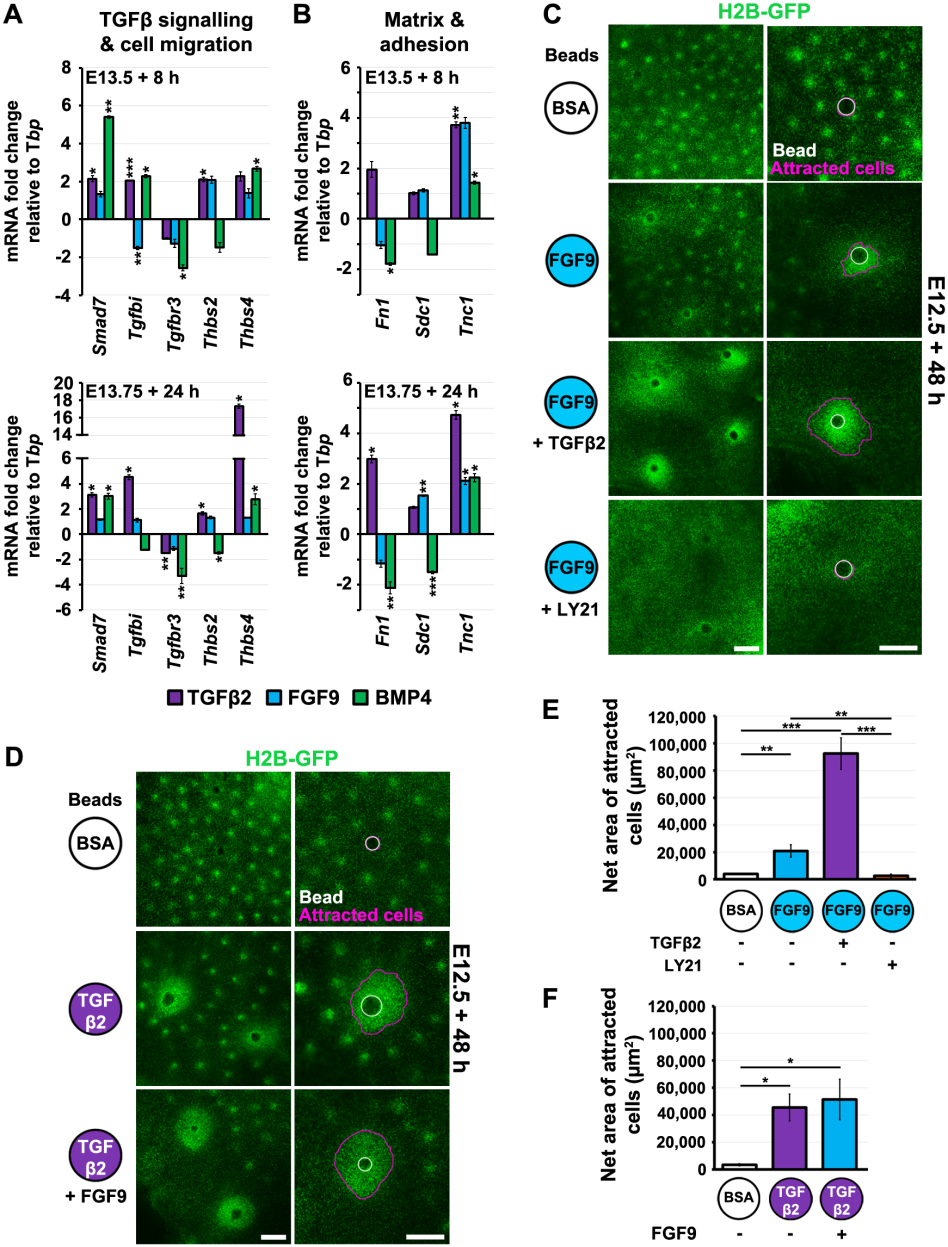
TGFβ enhances cell attraction to focal FGF sources. (A, B) Quantitative reverse transcription polymerase chain reaction (qRT-PCR) of E13.5 or E13.75 (with condensates) skins treated with transforming growth factor (TGF) β2, fibroblast growth factor (FGF) 9, or bone morphogenetic protein (BMP) 4 for 8 or 24 h, respectively, followed by assessment of transcript abundance. TGFβ2 upregulates expression of genes associated with cell movement and the extracellular matrix. Statistical significance from control was calculated using a Student *t* test (*p < 0.05, **p < 0.01, ***p < 0.001). Error bars represent SEM from at least 3 independent experiments. (C) Cell aggregation at FGF9 beads in E12.5 TCF/Lef::H2B-green fluorescent protein (GFP) skin explants. TGFβ2 (100 ng/ml) or LY2109761 (25 μM) is present in the culture medium as indicated. TGFβ2 enhances aggregation at FGF9 beads, while LY2109761 suppresses cell accumulation. (D) FGF9 presence in culture medium does not detectably increase cell recruitment to TGFβ2 beads. (E, F) Quantification of areas of high cell density around FGF9- or TGFβ2-coated beads under conditions as indicated. Statistical significance was calculated using Student *t* tests (*p < 0.05, **p < 0.01, ***p < 0.001). Error bars represent SEM of at least 3 independent experiments. Scale bars: 250 μm. The raw numerical values (for A, B, E, and F) can be found in S4 Data.

Having defined restricted TGFβ as a required self-attractive factor in mesenchyme-only patterning, we sought its role in an interplay between normal patterning and mesenchymal self-organisation. In addition to its function as a direct mesenchymal chemoattractant (Fig 6E) [61], TGFβ signalling also influences cell migration and cell–matrix interactions through modulation of gene expression to create an environment conducive to cell migration [62, 63]. We set out to identify in developing skin whether TGFβ regulates the expression of genes known to be associated with cell migration, adhesion, and matrix composition in other tissues [64–68]. We compared responses to TGFβ in both E13.5 and E13.75 skin with those elicited by similar FGF and BMP treatments, 2 signalling pathways we previously found to promote or antagonise dermal cell aggregation, respectively (Figs 2F and 3). We identified TGFβ regulation of genes encoding the cell migration modulators transforming growth factor beta induced (*Tgfbi*) and thrombospondin family members (*Thbs2* and *Thbs4*), as well as the matrix components Fibronectin (*Fn1*), Syndecan-1 (*Sdc1*), and Tenascin-C (*Tnc*) (Fig 7A and 7B), suggesting that TGFβ alteration of cell–matrix interaction may contribute to its aggregative effect. The TGFβ gene regulatory effects are broadly distinct from FGF-elicited responses, despite both signals sufficing to stimulate dermal cell aggregation at their local sources, while BMP suppresses *Fn1* and *Sdc1* expression and potentially downmodulates TGFβ signalling through induction of inhibitory *Smad7* and suppression of the TGFβ type III receptor (*Tgfbr3*), a known enhancer of TGFβ2 signalling [69].

These gene expression changes related to cell–matrix interactions and movement suggested that TGFβ may play a general role in promoting cell migration. To assess whether TGFβ could promote cell attraction to placodes, as indicated by the prominent condensates it induces during patterning (Fig 6B), we tested the effect of generalised TGFβ availability on directed movement to FGF sources, where loaded beads replicate the local source of FGF produced in a placode. We found that the extent of mesenchymal cell attraction to FGF-coated beads was strongly increased by the presence of TGFβ2 in the culture medium. Conversely, cell attraction to FGF beads was greatly diminished by TGFβ signal suppression (Fig 7C and 7E). We detected no reciprocal effect of ubiquitously available FGF on cell recruitment to local TGFβ2 (Fig 7D and 7F). Thus, TGFβ generates an environment conducive to efficient cell recruitment at FGF sources, explaining the phenomenon of delayed dermal condensate formation when this signal is suppressed.

## Discussion

Embryonic pattern formation proceeds rapidly; periodic arrangements of limb digits arise within approximately 16 h [70] and hair follicles in approximately 10 h [22]. By focussing on short-lived mRNAs, we have identified a network of interactions between the BMP, FGF, and WNT pathways capable of breaking symmetry to produce a periodic pattern. In this system, WNT signalling acts to stimulate expression of genes denoting placode fate (*Dkk4*, *Bmp2*, *Fgf20*), while BMP and FGF signalling inhibit their expression. We note that the half-lives of the proteins encoded by components of the network would also need to be relatively short to achieve rapid pattern formation. The many mRNAs with long half-lives that become preferentially expressed in the placodes, including *Ctnnb1*, *Wnt10a*, *Wnt10b*, and *Edar*, may act to stabilise the rapidly emerging pattern and promote hair follicle growth, as previously shown for EDAR function [22, 25, 40].

Following definition of the epidermal placode arrangement, dermal cells are locally recruited to assemble a condensate. Previously, a mesenchymal cell-sorting mechanism for hair follicle patterning was proposed based on genetic correlations between hair follicle number and size [71], conceptually similar to the well-studied and diverse colour patterns on fish skin arising from sorting of distinct mesenchymal lineages [15]. Though there is abundant heterogeneity of dermal mesenchymal cells that could be exploited to achieve such a mechanism [72], our quantitative analyses do not support the operation of such a system and instead show that the cells ultimately forming the condensate are those located in its immediate proximity (Fig 2). The local movement of the dermal mesenchymal cells is thus similar to the short-range movements that act to construct the epithelial placode [20].

In addition to their contribution to pattern formation through rapid gene regulatory interactions, the BMP and FGF pathways also influence mesenchymal cell behaviour. FGF, normally produced locally at the placode, directs mesenchymal clustering, while BMP suppresses aggregation. By mimicking the hair follicle microenvironment of high-FGF and low-BMP signalling, we identify a latent potential of dermal mesenchyme to self-organise, yielding a stable periodic pattern of cell aggregates in the absence of signalling directives from the epidermis. This demonstrates that dermal condensates do not require spatially restricted signals to form. Rather, the widespread BMP activity in the skin prior to hair follicle patterning, together with the slow rate of dermal self-organisation, subordinates the mesenchyme’s patterning potential to reaction–diffusion patterning in the epidermis. Our identification of a mesenchyme-only patterning condition is, in structural terms, reciprocal to that arising from *Fgf20* deficiency, which results in epidermal placode formation without accompanying mesenchymal condensates [27]. Thus, epidermal and dermal patterning are separable self-organising systems, coupled through epidermal FGF-guided mesenchymal cell attraction with TGFβ potentiation of this accumulation.

Mechanistically, however, these epidermis-only and mesenchyme-only patterns arise through very different mechanisms. Epidermis-only patterning in the *Fgf20* mutant is achieved through focally restricted signals, agreeing with the majority of patterning signals being produced in this tissue layer (see Fig 1D and S1 Table), while it is motile cells that determine the dermis-only pattern. The expansion of placode identity upon suppression of TGFβ signalling (Fig 6B), similar to that of the unresolved prepattern (Fig 2E), may be a secondary result of delayed condensate formation together with its accompanying inhibitors (BMP4 and DKK1) failing to define the placode edges.

Our findings of dermal condensate formation in the absence of an epidermal prepattern are consistent with modes of tissue patterning in which moving mesenchymal cells themselves are agents of pattern formation. These models do not require interacting diffusible signals and such mechanisms integrate tissue morphogenesis and patterning as part of a single process, rather than occurring in sequence, as in the Turing system [4, 8, 9, 73]. An aggregative potential of dermal papilla cells, the product of the embryonic dermal condensate, has been noted [74] and may not be unique to the skin, as spontaneous condensation of mesenchymal stem cells from a range of organs has been identified, given suitable culture substrates in vitro [75]. Beyond the formation of simple condensates, spatially organised aggregates of embryonic limb bud mesenchymal cells arise spontaneously in culture [76], with a suggested role for TGFβ2 driving this process for mesenchymal cells cultured in isolation from epithelium [77]. These observations may suggest a broad role for TGFβ in driving mesenchymal morphogenesis, whether spatial organisation is guided by a prepattern or occurs de novo through cell movement.

**Fig 8:**
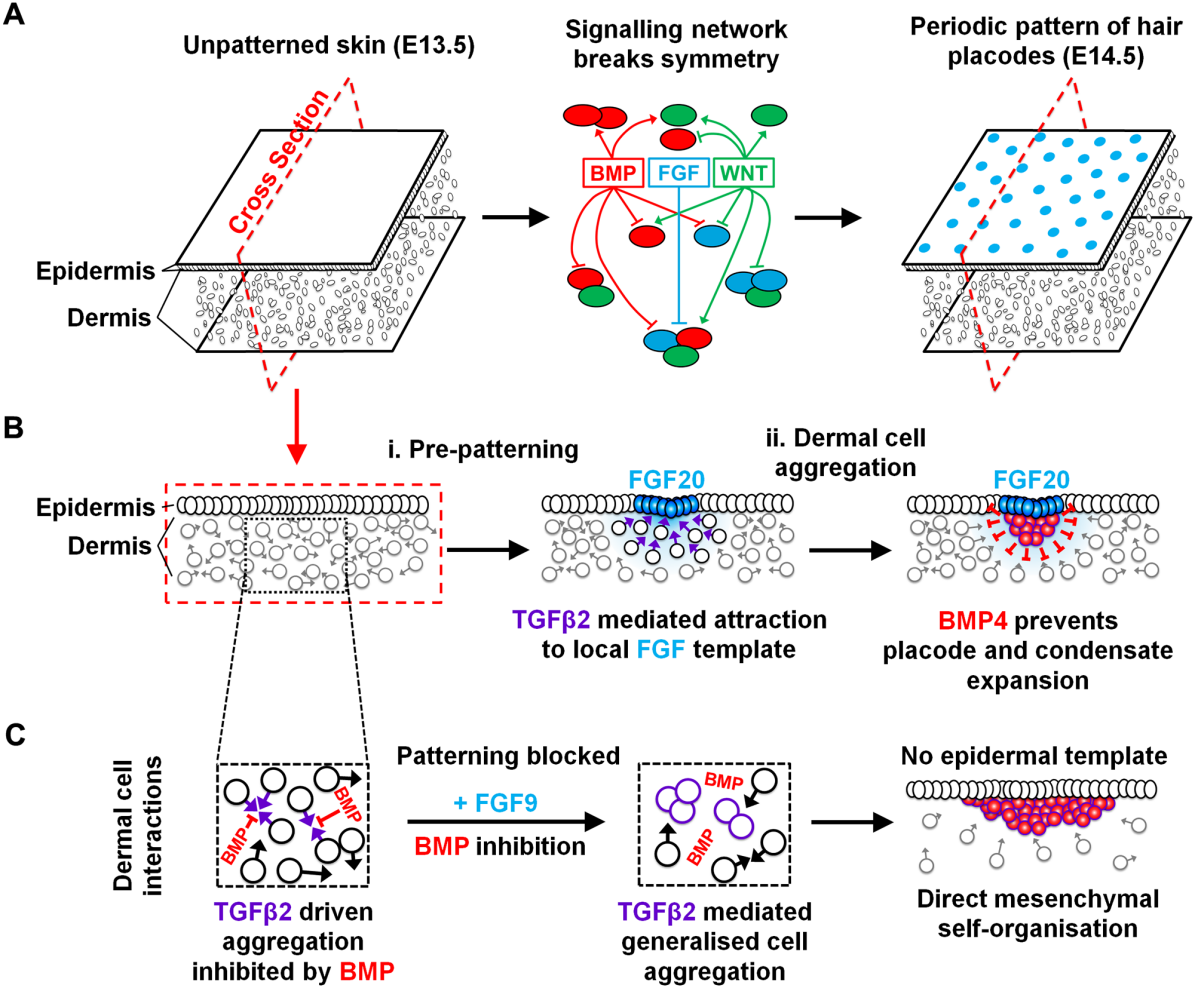
Overlaying of reaction-diffusion signalling and dermal condensation mechanisms. (A) A signalling network including bone morphogenetic protein–fibroblast growth factor–wingless-related integration site (BMP–FGF–WNT) interactions rapidly produces a periodic prepattern of hair placodes with high WNT/β-catenin activity. (B) FGF20 production by placodes leads to local dermal cell attraction and condensate formation, facilitated by widespread transforming growth factor (TGF) β activity. BMP production inhibits expansion of placode gene expression and further dermal cell attraction. (C) In the absence of epidermal patterning, no localised attractant signals from placodes are present, and dermal BMP signalling is uniform. TGFβ stimulates dermal cell–cell attraction, which is inhibited by BMPs. Suppression of BMP signalling permits TGFβ-driven mesenchymal patterning in the absence of a functioning epidermal reaction–diffusion network. Condensate expansion is restricted by mesenchymal cell depletion from the surrounding dermis.

Together, these results support a model for hair follicle positioning and construction in which a reaction–diffusion system, operating largely in the epidermis [25, 27, 39], limits the locations at which mesenchymal organisation can occur by providing both local FGF direction and relief from BMP activity (Fig 8). Dermal cells respond to these sources of FGF, with widespread TGFβ2 generating an environment promoting entry into condensates according to the FGF gradients. As it forms, the condensate produces BMP4, thereby restricting further condensate and placode expansion. Ultimately, the signals operating in reaction-diffusion patterning also serve to modulate mesenchymal behaviour to direct, inhibit, or stimulate mesenchymal condensation, such that, under FGF^Hi^BMP^Lo^ conditions, the components of the normal patterning network are recast to achieve mesenchyme-only patterning. Thus, we conclude that the dermal mesenchyme does possess pattern-generating ability but that this is preceded by a rapid, primarily epidermal, pre-patterning system that acts to specify the restricted locations at which mesenchymal organisation is permitted.

The rationale for the existence of 2 distinct routes to achieve periodic patterning during hair follicle formation may lie in a shifting balance between these 2 potentials in the waves of hair follicles that form at different developmental stages. Thus, it may be that the pattern of secondary or tertiary hair follicles is defined by mesenchymal cell behaviour to a greater extent than the primary follicles focussed upon here. Alternatively, the existence of 2 periodicity generators may be a remnant of the evolutionary history of skin development, as it has been suggested that turnover of patterning mechanisms can occur during the course of evolution [78, 79] and that direct cell-driven patterning systems tend to become captured by signal-based pre-patterning systems [80]. Our work demonstrates experimentally such a superimposition of different patterning mechanisms, with 1 potential for self-organisation being suppressed and subjected to instruction by another.

## Materials and methods

### Ethics statement

All animal work was conducted under approval of the Animal Welfare and Ethical Review Body (AWERB) at The Roslin Institute, University of Edinburgh, and by the United Kingdom Home Office in accordance with the Animals (Scientific Procedures) Act 1986. Euthanasia was carried out according to Schedule 1 of the Animals (Scientific Procedures) Act 1986.

### Animals

Mice were on the FVB/N background, apart from those employed in time-lapse and static imaging of cell rearrangement, which were offspring of a cross between male hemizygous TCF/Lef::H2B-GFP mice [43] on a mixed C3H/C57 genetic background and female FVB/N mice. Noon on the day of discovery of the vaginal plug was designated day 0.5 of development. Skins were examined for pre-existing signs of hair morphogenesis and not used if these were detected for experiments designated to start prior to follicle morphogenesis. Embryos were harvested in high-glucose Dulbecco’s modified Eagle’s medium (DMEM) (Sigma) supplemented with 1% penicillin/streptomycin (Gibco, Life Technologies) and kept on ice for short periods.

### Skin organ culture and treatment

For the qRT-PCR experiments used in the network derivation, dorsal skin explants were halved along the midline to generate treated and control halves from each embryo. Skin halves were placed onto a cellulose filter (Millipore, pore size 0.45 μm). The tissue was then submerged in DMEM supplemented with 2% FBS (complete DMEM) in a centre-well dish (Falcon) and incubated at 37°C and 5% CO_2_ for 6 h. For the TGFβ2, FGF9, and BMP4 experiments, whole dissected skins were cultured in complete DMEM containing the required recombinant protein for either 8 h or 24 h.

For longer-term culture, whole skin explants were dissected and mounted on filters as described above, submerged in complete DMEM, and supported by a metal grid in a centre-well dish. E13.5 explants were cultured for 27 h unless stated otherwise. For experiments extending beyond 27 h, medium was replaced every 24 h.

Epidermal–dermal separations for RNA-seq and qRT-PCR experiments were performed by incubating skin samples with 2 mg/mL Dispase II (Gibco) at 37°C for 10 minutes. For imaging, epidermal–dermal separations were performed by incubating skin in 10 mM EDTA/PBS for 25 minutes at 37°C followed by 20 minutes in PBS at RT prior to separation with fine forceps. For counterstaining, samples were fixed in 4% PFA, washed several times in PBS/0.1% Tween 20 (PBST), treated with 20 μg/ml proteinase K for 3 minutes, washed in PBT, incubated with 100 μg/ml RNase for 20 minutes, washed again with PBT and then stained with 1/2000 diluted propidium iodide (PI) solution (Life Technologies) for 5 minutes. Following staining, samples were washed in PBT and mounted in Prolong Gold (Life Technologies).

Recombinant FGF7 (mouse), FGF9 (mouse and human), BMP4 (mouse), and TGFβ2 (human) were from R&D Systems. LDN193189 (Stemgent), CHIR99021 (Axon biochem), IWR-1 (Tocris), DAPT (Bio-Techne), LY2109761 (Cambridge Biosciences), Pertussis toxin (Tocris), Imatinib Mesylate (Sigma Aldrich), and SU5402 (Sigma Aldrich) were reconstituted according to the manufacturer’s recommendations. We did not detect any significant effect from treatment with recombinant FGF20 protein obtained from 2 commercial suppliers on either placode pattern formation, skin development, or on expression of the direct FGF pathway target gene *Etv5*.

### qRT-PCR

Total RNA was isolated from skin explants using Tri Reagent (Sigma) and treated with RQ1 DNAse (Promega) to remove contaminating genomic DNA. cDNA was synthesised from total RNA using random primers and Superscript III reverse transcriptase (Roche) in a 20-μl reaction. Reactions were diluted 20-fold and had 3 μl used as a template for each qRT-PCR. Each reaction was performed in a 20-μl volume using Universal SYBR Green Master Mix (Roche) containing Rox reference dye. Reactions were performed in triplicate, with at least 3 biological replicates used to determine each data point. Relative expression levels were determined from cDNA dilution standard curves and normalised to *Tbp* (or *Capzb* for half-life determination). Oligonucleotide sequences used are given in S1 Supporting Methods.

### Genome-wide mRNA half-life determination

See S1 Supporting Methods for Actinomycin D dose determination and treatment, sample quality control and processing, RNA-sequencing, analysis, and qRT-PCR validation.

### In situ hybridisation

Skin explants were fixed overnight in 4% PFA at 4°C. Samples were dehydrated into 100% methanol, bleached in 6% H_2_O_2_, then rehydrated and treated with 20 μg/mL proteinase K. After postfixing in 4% PFA containing 0.2% glutaraldehyde for 20 minutes, skin explants were hybridised with probe at 60°C overnight in 50% formamide, 5 X saline sodium citrate (SSC), 1% SDS, 50 μg/mL heparin, and 50 μg/mL yeast RNA in diethyl pyrocarbonate (DEPC)-treated H_2_O. Samples were washed to remove unbound probe and signal detected using an alkaline phosphatase sheep antidigoxigenin antibody (Roche, 1:1,000 dilution) and 5-bromo-4-chloro-3'-indolylphosphate/nitro-blue-tetrazolium (BCIP/NBT) colour reaction (Sigma).

### Immunoblotting and immunohistochemistry

For immunohistochemistry, samples were fixed in 4% PFA, embedded in 0.12 M sodium phosphate buffer/7.5% gelatin/15% sucrose, and cryosectioned. Sections were rehydrated in PBS at 37°C for 30 minutes, briefly washed with tris-buffered saline containing 0.01% Tween 20 (TBST), then incubated in blocking buffer (TBST containing 5% heat-treated sheep serum and 1% BSA) for 1 h at RT before overnight incubation at 4°C with primary rabbit antibodies (1:200 mouse antiactive beta catenin (Millipore #05–665) or rabbit antiphosphorylated SMAD2 (pSMAD2) (Cell Signalling Technologies #3108) diluted in blocking buffer. Samples were washed in TBST, then incubated with fluorescent secondary antibodies (1:500 Life Technologies) in blocking buffer for 1 h at RT. Samples were washed with TBST, counterstained with DAPI (Sigma) and mounted in Prolong Gold (Life Technologies). Sections were imaged using a Zeiss LSM710 confocal microscope (Carl Zeiss).

For immunoblotting, protein was extracted from skin samples using radioimmunoprecipitation assay (RIPA) lysis buffer (Santa Cruz Technologies) and a handheld homogeniser. Protein samples were diluted in NuPage LDS sample buffer (Life Technologies), separated by gel electrophoresis on 4%–12% Bis-Tris NuPage precast gels under denaturing conditions and transferred to nitrocellulose membrane (Amersham). Membranes were blocked in 5% milk/TBST for 1 h at RT, followed by overnight incubation at 4°C in 5% BSA/TBST containing primary antibody (1:1,000 mouse anti-active β-catenin [Millipore #05–665], 1:3000 rabbit anti-β-catenin [BD biosciences #610153], 1:1,000 rabbit anti-phospho SMAD 2/3 [Cell Signalling Technologies #8828], 1:1,000 rabbit anti-SMAD2 [Cell Signalling Technologies #5339], or 1:3,300 mouse anti-γ tubulin [Sigma Aldrich #T6557]). Following primary antibody incubation, membranes were washed in TBST and incubated for 1 h at RT in 5% milk/TBST containing horseradish peroxidase (HRP)-conjugated species-specific secondary antibodies (Dako). Membranes were washed several times with TBST before detection with the Novex ECL chemiluminescent substrate reagent kit (Life Technologies) and developed on ECL Film (Amersham).

### Bead experiments

Affi-Gel Blue Gel beads (Bio-Rad) were washed twice in PBS and incubated in either 100 μg/ml recombinant human FGF9, 100 μg/ml recombinant human TGFβ2, or 100 μg/ml BSA diluted in PBS for at least 2 h at RT or overnight at 4°C. Beads were placed onto a nitrocellulose Millipore filter (pore size 0.45 μm), and dissected E12.5 TCF/Lef::H2B-GFP dorsal skin explants were manoeuvred and placed on top of the beads, dermis side down. Skins were then imaged, as described in S1 Supporting Methods, over a period of 48–72 h.

### Live imaging and microscopy

E13.5 TCF/Lef::H2B-GFP dorsal skin explants were dissected and imaged as described previously using a custom imaging chamber [81] with the following modifications. Instead of using a central imaging clip, the entire base of the chamber was filled with 1% (weight/volume) agarose in PBS. Dissected embryonic skin was mounted dermis-side down onto a black nitrocellulose filter membrane (Millipore) with a 45 μm pore size. The membrane was subsequently placed onto the agarose block, and a lummox membrane (Greiner) was clamped across it with an o-ring such that the skin was sandwiched between the 2 membranes. The imaging chamber was filled with DMEM without phenol red containing 4,500 mg/L glucose, 2% FBS, 1% penicillin/streptomycin, and 0.584 g/l L-glutamine. Images were captured with a 20X objective using a Nikon A1R inverted confocal microscope in a heated chamber supplied with 5% CO_2_ in air. Bead and FGF9/LDN193189 combination experiments were performed using a Zeiss Live Cell Observer and are described in S1 Supporting Methods.

### Image analysis

All image analysis tasks were performed using custom written macros for the Fiji [82] distribution of ImageJ, an open-source image analysis package based on NIH Image [83]. The source code is available through GitHub (https://github.com/richiemort79/cell_patterning). In order to track cell behaviour in an unbiased manner, maximum intensity Z-projections of time-lapse sequences were drift-corrected and cropped to include the area of the final condensate (of varying size) with approximately 100 μm of space surrounding each. A window of at least 1,500 minutes that incorporated condensate formation was considered. At least 50 cells per condensate from at least 4 independent skins were then selected at random prior to condensate formation and tracked manually until they either entered a condensate or the video ended.

A cell was deemed to acquire a ‘condensate identity’ if, at the end of the tracking period, it was incorporated within this structure; for analysis purposes, tracking was halted on follicle entry. Cells that did not enter the condensate were termed ‘intercondensate.’ Visual analysis of these cells up to a later time point was also performed to ensure that these cells did not become part of the condensate at a later stage.

## Supporting information

S1 Fig Characterisation of the TCF/Lef::H2B-GFP marker in developing skin. (A) E13.5 (prior to hair patterning) dorsal back skin from TCF/Lef::H2B-GFP mice demonstrates GFP expression specifically in dermal cell nuclei thereby allowing mesenchymal cells to be visualised during hair follicle morphogenesis. (B) E13.5 TCF/Lef::H2B-GFP dorsal skin explant cultured for 27 hrs and imaged for GFP (left). Epidermis and dermis separated and counterstained with propidium iodide (PI) (right). In the epidermis, signal has appeared in the placodes, while the dermis shows widespread nuclear GFP, which reveals the aggregated cells comprising the dermal condensates. (C) Dose effects of modulators of BMP, FGF and WNT signalling on periodic patterning assessed by *Dkk4* expression. Scale bars as indicated.
(TIF)

S2 Fig FGF7 treatment inhibits epidermal placode and dermal condensate formation. E13.5 TCF/Lef::H2B-GFP dorsal skin explants were treated with increasing concentrations of FGF7 for 27 hrs, GFP expression analysed and *Dkk4* detected by *in situ* hybridisation. Scale bars: 250 μm.
(TIF)

S3 Fig Determination of mRNA half-lives in embryonic epidermis and dermis. (A) Schematic of experimental approach to define mRNA half-lives and derive a network of interactions between BMP, FGF and WNT pathways. Briefly, paired skin halves were either processed for RNA isolation immediately or else cultured in Actinomycin D to inhibit RNA synthesis for a set period, then processed for RNA isolation. Epidermal and dermal layers were isolated and RNA-sequencing performed on each layer separately. The ratio of RNA species abundance between the paired skin samples was used to calculate t_1/2_. Candidate genes were identified based on half-life and a gene regulatory network was derived through systematic experimental and mathematical approaches. (B) [^3^H] cytidine incorporation assay to determine Actinomycin D dose sufficient to block transcription in cultured embryonic skin. Each point represents the [^3^H] cytidine incorporation per A260 unit for a single skin sample. (C) Transcripts from RNA-seq experiments expressed above threshold level in epidermis or dermis, grouped according to half-life. Of the 37,315 transcripts detectable by RNA-seq, 11,970 were detected in the epidermis and 12,597 in the dermis. (D) qRT-PCR validation of selected mRNA half-lives from RNA-seq data. E13.5 dorsal skin explants were treated with Actinomycin D for 30, 60 or 120 minutes. Relative expression was calculated by normalising the expression at each experimental time to the expression of that gene at time 0. The raw numerical values for (B-D) can be found in S5 Data. (E) Table of the candidates validated by qRT-PCR in (D) alongside the original values from RNA-seq. (F) Matrix of signalling pathway interactions derived from network in Fig 1E used for mathematical analysis (S1B and S1C Appendix). All genes in the network were grouped into one of five species: BMP (*Bmp2*, *Bmp4*, *Rgma*), WNT (*Wnt9a*, *Ror2*, *Fzd10*), WNT inhibitor (*Dkk1* and *Dkk4*), BMP inhibitor (*Bambi* and *Nog*) or FGF (*Fgf20*, representing the most strongly regulated family member). Inhibition is represented by a – sign and stimulation by a + sign.
(TIF)

S4 Fig FGF suppresses epidermal WNT/β-catenin signalling. (A) qRT-PCR detecting *Etv5* expression in epidermal and dermal fractions of skin cultured with FGF9 for 6 hrs. Gene expression is induced in both tissue compartments. Error bars represent s.e.m. of three independent skins. Statistical significance from control was calculated using a Student t test, (**p < 0.01). The raw numerical values can be found in S1 Data. (B) Immunodetection of active β-catenin (ABC) in cryosections from dorsal skin explants treated with FGF9 or the β -catenin activator CHIR99021 for 6 hrs. Tissue layers are demarcated by dotted lines. Ep. = Epidermis, De. = Dermis. Scale bar as annotated. (C) Immunoblot determination of ABC in isolated epidermis of skin explants treated with FGF9 or CHIR99021 for 6 hrs. Total β-catenin and γ-tubulin were used as loading controls.
(TIF)

S5 Fig Mesenchymal self-organisation is not stage dependent. (A) E12.5 TCF/Lef::H2B-GFP dorsal skin explants cultured with either FGF9 (1 μg/ml), LDN193189 (LDN) (10 μM) or both for 48 hrs show a periodic pattern of cellular aggregates and lack *Dkk4* foci. Scale bar: 250 μm. (B) GFP signal in E13.5 TCF/Lef::H2B-GFP dorsal skin explants cultured in FGF9 and LDN for 72 hrs. Arrows indicate primary hair follicle primordia, arrowheads show secondary follicle primordia. Application of FGF and suppression of BMP signalling permits the formation of mesenchymal aggregate patterns at each stage, and secondary follicle primordia are not inserted independently between mesenchymal patterns in FGF^Hi^BMP^Lo^ conditions. Scale bar: 250 μm.
(TIF)

S6 Fig Mesenchymal self-organisation produces autonomously stable cell aggregates. E12.5 TCF/Lef::H2B-GFP skin explants were cultured in the presence of FGF9 (1 μg/ml), LDN193189 (LDN) (10 μM) or both for 27 hours, then imaged and this condition was either reapplied (A) or removed (B) and culture continued for a further 24 hours. Mesenchymal condensations formed in the FGF^Hi^BMP^Lo^ conditions are stable and continue to resolve after removal of FGF9 and LDN. Scale bar: 250 μm.
(TIF)

S7 Fig Mesenchymal self-organisation is slower than epithelium-guided condensate formation. E12.5 TCF/Lef::H2B-GFP skin explants were cultured over a period of 48 hrs and imaged at time intervals as labelled. FGF^Hi^BMP^Lo^ pattern formation is slower to resolve than control condensate formation. Scale bar: 1 mm.
(TIF)

S8 Fig Mesenchymal condensate formation is resistant to modulation of G-protein, PDGF and NOTCH signalling pathways. FGF^Hi^BMP^Lo^ condensations are formed in the presence of pertussis toxin (PTX, a G-protein inhibitor), imatanib mesylate (IM, a PDGFR inhibitor) and DAPT (a NOTCH inhibitor). Scale bars: 250 μm.
(TIF)

S9 Fig Delayed follicle morphogenesis in the presence of LY2109761. (A) Images of E13.5 TCF/Lef::H2B-GFP skin cultured for 48 hours with or without TGFβ inhibition by LY2109761 (25 μM). Hair follicle primordia in control skin have formed a dermal papilla and polarised from anterior to posterior (inset), while TGFβ inhibited samples have not progressed to this stage. Scale bar: 250 μm. (B) Haematoxylin stained sections of skin samples in panel (A). Placode downgrowth is delayed in the LY2109761 condition, consistent with the reported phenotype of the *Tgfb2* mutant. Scale bar: 100 μm.
(TIF)

S1 Table Transcript abundance in E13.5 epidermis and dermis, and mRNA half-life (t_1/2_), for candidate genes that fulfil selection criteria. Transcript names are in coloured text according to the pathway in which they operate: BMP (green), WNT (red), FGF (blue).
(XLSX)

S2 Table Full gene list of read counts and calculated transcript half-lives normalised to *Capzb* from RNA-seq of Actinomycin-D treated E13.5 dermis and epidermis. Results from experiment outlined in S2A Fig. R^2^ values are curve fit to exponential function.
(XLSX)

S3 Table List of extracellular molecules within the BMP, FGF and WNT pathways. (XLSX)

S1 Data Data pertaining to Fig 1D & S4A Fig. (XLSX)

S2 Data Data pertaining to Figs 2B–2D and 5B–5E. (XLSX)

S3 Data Data pertaining to Fig 3B, 3C, 3D and 3F. (XLSX)

S4 Data Data pertaining to Fig 7A, 7B, 7E and 7F. (XLSX)

S5 Data Data pertaining to S3B–S3D Fig. (XLSX)

S1 Appendix Network construction, mathematical methods, and particle image velocimetry analyses. (DOCX)

S1 Supporting Methods Additional experimental methods and materials. (DOCX)

S1 Video Live cell imaging of hair follicle formation. Confocal time-lapse video of cultured E13.5 TCF/Lef::H2B-GFP dorsal skin explant undergoing pattern formation. Scale bar: 100 μm.
(AVI)

S2 Video Live cell imaging of dermal condensate formation. Time-lapse video of cultured E13.5 TCF/Lef::H2B-GFP dorsal skin explant undergoing pattern formation. Randomly selected cells were tracked for each individual follicle analysed. The boundary of the final condensate is indicated by the white circle. Cells which ultimately enter and form the condensate are indicated as yellow tracks while those that remain outside as intercondensate cells appear as red tracks. Scale bars as annotated.
(MP4)

S3 Video Dermal cells are attracted to local sources of FGF. Time-lapse imaging of E12.75 TCF/Lef::H2B-GFP dorsal skin explants cultured in the presence of either FGF9 loaded or control BSA beads. Scale bars as annotated.
(M4V)

S4 Video Comparison of pattern formation in FGF^Hi^BMP^Lo^ conditions. Live cell imaging of E13.5 TCF/Lef::H2B-GFP dorsal skin explants cultured in FGF9 (1 μg/ml) and LDN193189 (LDN) (10 μM) either alone or in combination. Treatment with FGF9 and LDN results in labyrinthine patterning of dermal condensates. Scale bars as annotated.
(M4V)

S5 Video Pattern formation in FGF^Hi^BMP^Lo^ conditions. Confocal time-lapse video of cultured E13.5 TCF/Lef::H2B-GFP dorsal skin explant treated with FGF9 (1 μg/ml) and LDN 193189 (LDN) (10 μM) and imaged undergoing pattern formation.
(AVI)

## Abbreviations

BCIP: 5-bromo-4-chloro-3'-indolylphosphate
BMP: bone morphogenetic protein
BSA: bovine serum albumin
DAPI: 4’6-diamidino-2-phenylindole dihydrochloride
DEPC: diethyl pyrocarbonate
DKK: Dickkopf
DMEM: Dulbecco’s modified Eagle’s medium
EDAR: ectodysplasin A receptor
FGF: fibroblast growth factor
GFP: green fluorescent protein
HRP: horseradish peroxidase
NBT: nitro-blue-tetrazolium
NCAM: neural cell adhesion molecule
PDGF: platelet-derived growth factor
PI: propidium iodide
RIPA: radioimmunoprecipitation assay
SSC: saline sodium citrate
TBST: tris-buffered saline containing Tween
TGF: transforming growth factor
WNT: wingless-related integration site
