# Supplementary figures and images for: Hierarchical patterning modes orchestrate hair follicle morphogenesis
Source: PLoS Biol. 2017 Jul 11;15(7):e2002117. doi: 10.1371/journal.pbio.2002117 (PMC5507405; doi:10.1371/journal.pbio.2002117)

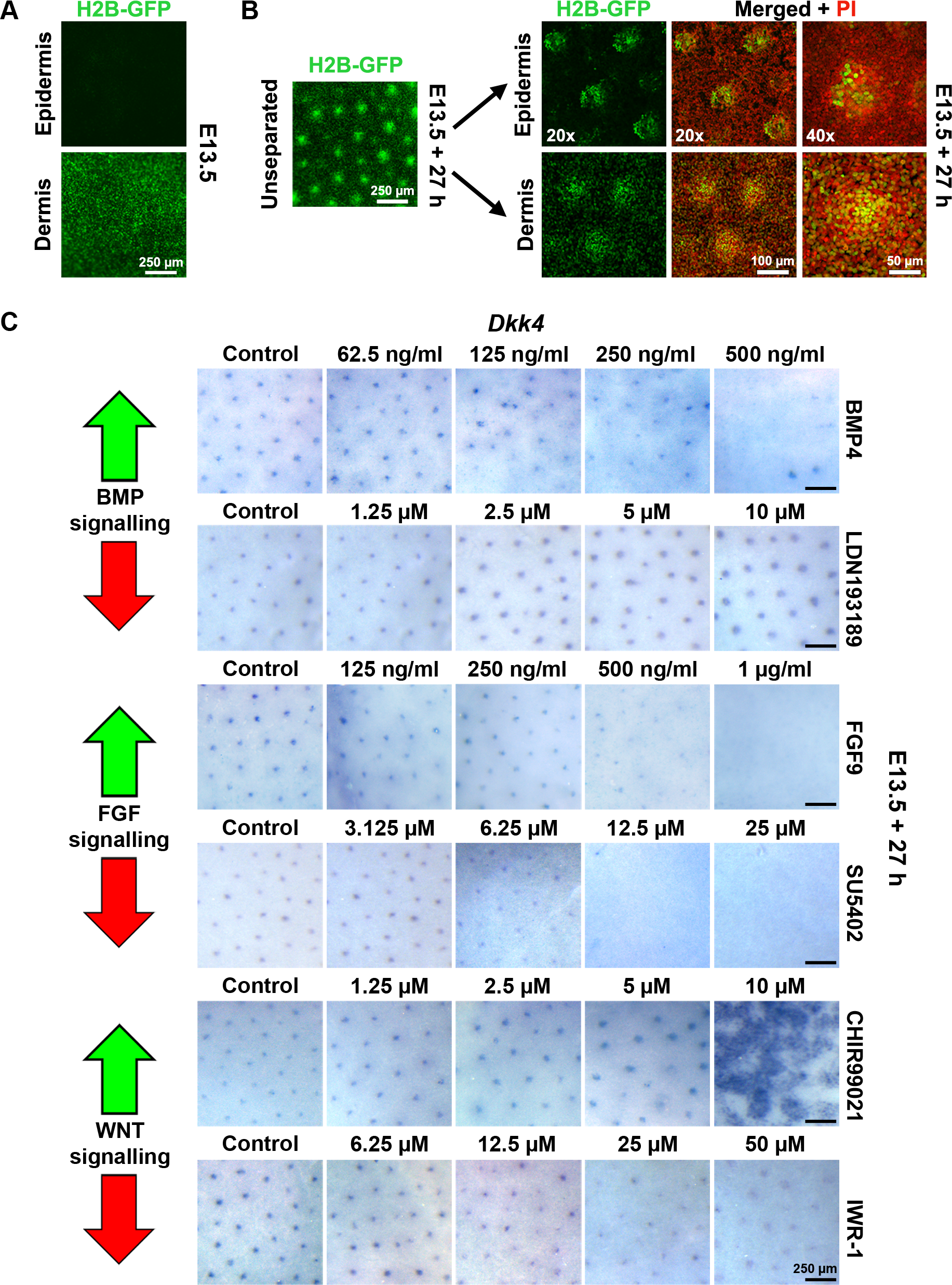

Supplement: S1 Fig — (A) E13.5 (prior to hair patterning) dorsal back skin from TCF/Lef::H2B-GFP mice demonstrates GFP expression specifically in dermal cell nuclei thereby allowing mesenchymal cells to be visualised during hair follicle morphogenesis. (B) E13.5 TCF/Lef::H2B-GFP dorsal skin explant cultured for 27 hrs and imaged for GFP (left). Epidermis and dermis separated and counterstained with propidium iodide (PI) (right). In the epidermis, signal has appeared in the placodes, while the dermis shows widespread nuclear GFP, which reveals the aggregated cells comprising the dermal condensates. (C) Dose effects of modulators of BMP, FGF and WNT signalling on periodic patterning assessed by Dkk4 expression. Scale bars as indicated. (TIF) [file pbio.2002117.s001.tif]

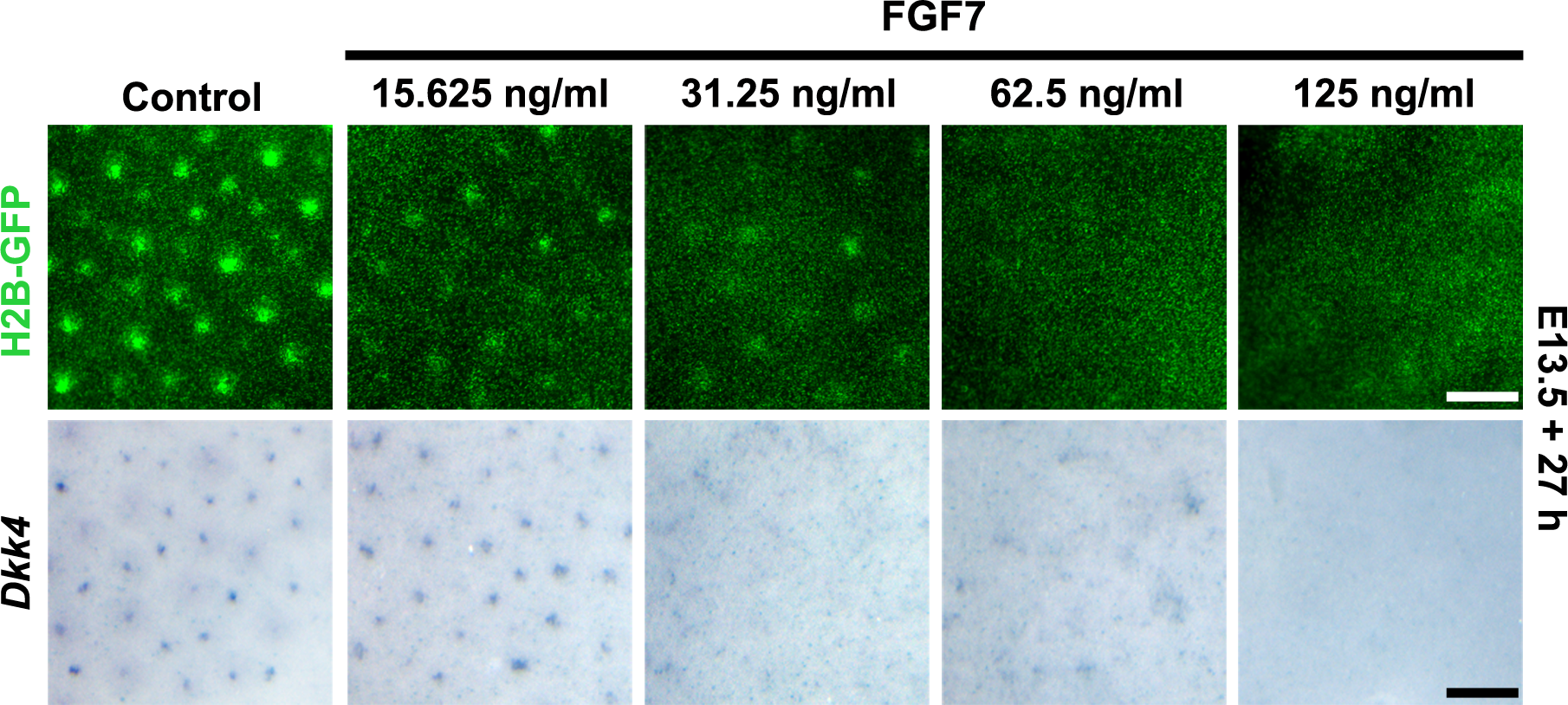

Supplement: S2 Fig — E13.5 TCF/Lef::H2B-GFP dorsal skin explants were treated with increasing concentrations of FGF7 for 27 hrs, GFP expression analysed and Dkk4 detected by in situ hybridisation. Scale bars: 250 μm. (TIF) [file pbio.2002117.s002.tif]

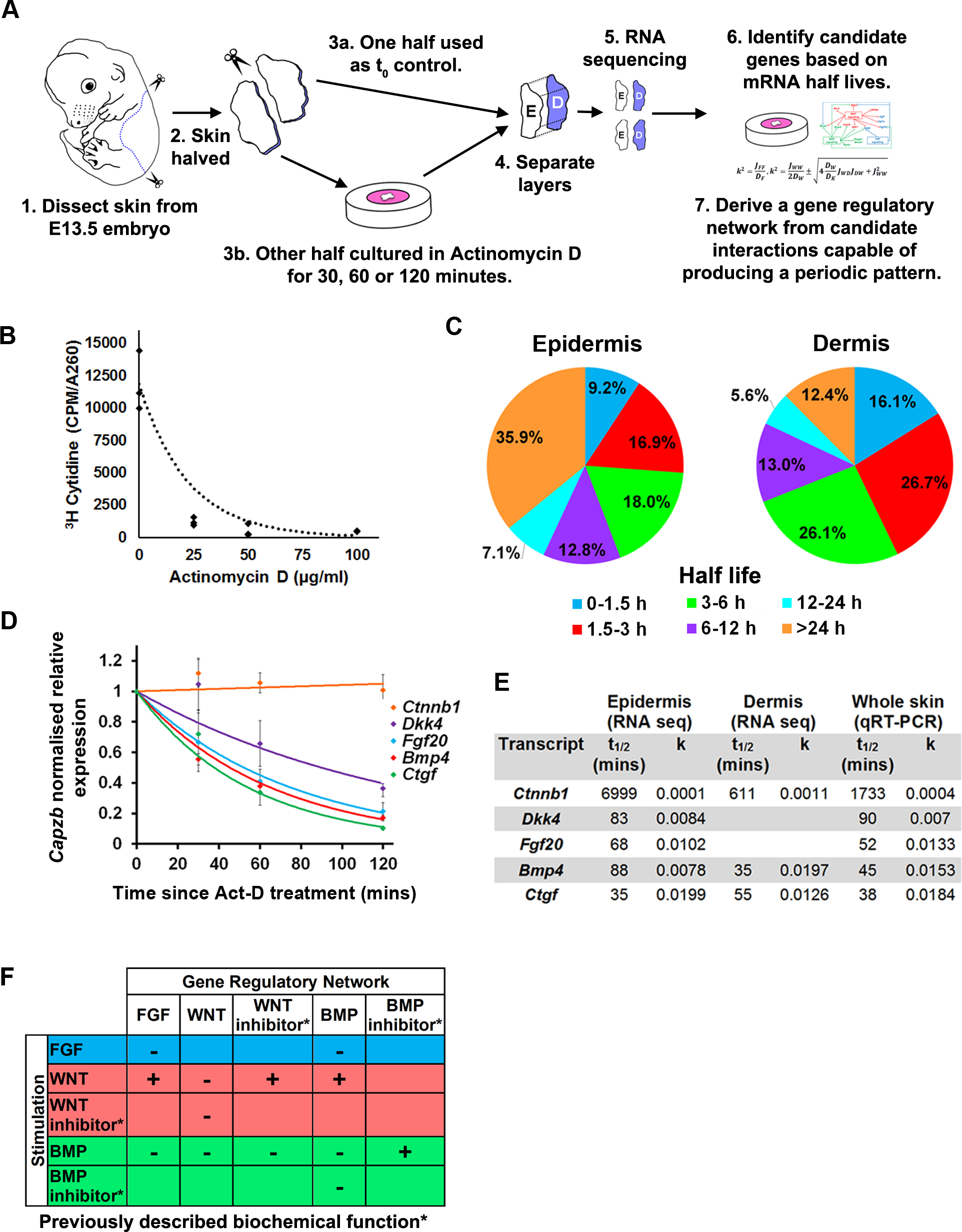

Supplement: S3 Fig — (A) Schematic of experimental approach to define mRNA half-lives and derive a network of interactions between BMP, FGF and WNT pathways. Briefly, paired skin halves were either processed for RNA isolation immediately or else cultured in Actinomycin D to inhibit RNA synthesis for a set period, then processed for RNA isolation. Epidermal and dermal layers were isolated and RNA-sequencing performed on each layer separately. The ratio of RNA species abundance between the paired skin samples was used to calculate t1/2. Candidate genes were identified based on half-life and a gene regulatory network was derived through systematic experimental and mathematical approaches. (B) [3H] cytidine incorporation assay to determine Actinomycin D dose sufficient to block transcription in cultured embryonic skin. Each point represents the [3H] cytidine incorporation per A260 unit for a single skin sample. (C) Transcripts from RNA-seq experiments expressed above threshold level in epidermis or dermis, grouped according to half-life. Of the 37,315 transcripts detectable by RNA-seq, 11,970 were detected in the epidermis and 12,597 in the dermis. (D) qRT-PCR validation of selected mRNA half-lives from RNA-seq data. E13.5 dorsal skin explants were treated with Actinomycin D for 30, 60 or 120 minutes. Relative expression was calculated by normalising the expression at each experimental time to the expression of that gene at time 0. The raw numerical values for (B-D) can be found in S5 Data. (E) Table of the candidates validated by qRT-PCR in (D) alongside the original values from RNA-seq. (F) Matrix of signalling pathway interactions derived from network in Fig 1E used for mathematical analysis (S1B and S1C Appendix). All genes in the network were grouped into one of five species: BMP (Bmp2, Bmp4, Rgma), WNT (Wnt9a, Ror2, Fzd10), WNT inhibitor (Dkk1 and Dkk4), BMP inhibitor (Bambi and Nog) or FGF (Fgf20, representing the most strongly regulated family member). Inhibition is represented by a [file pbio.2002117.s003.tif]

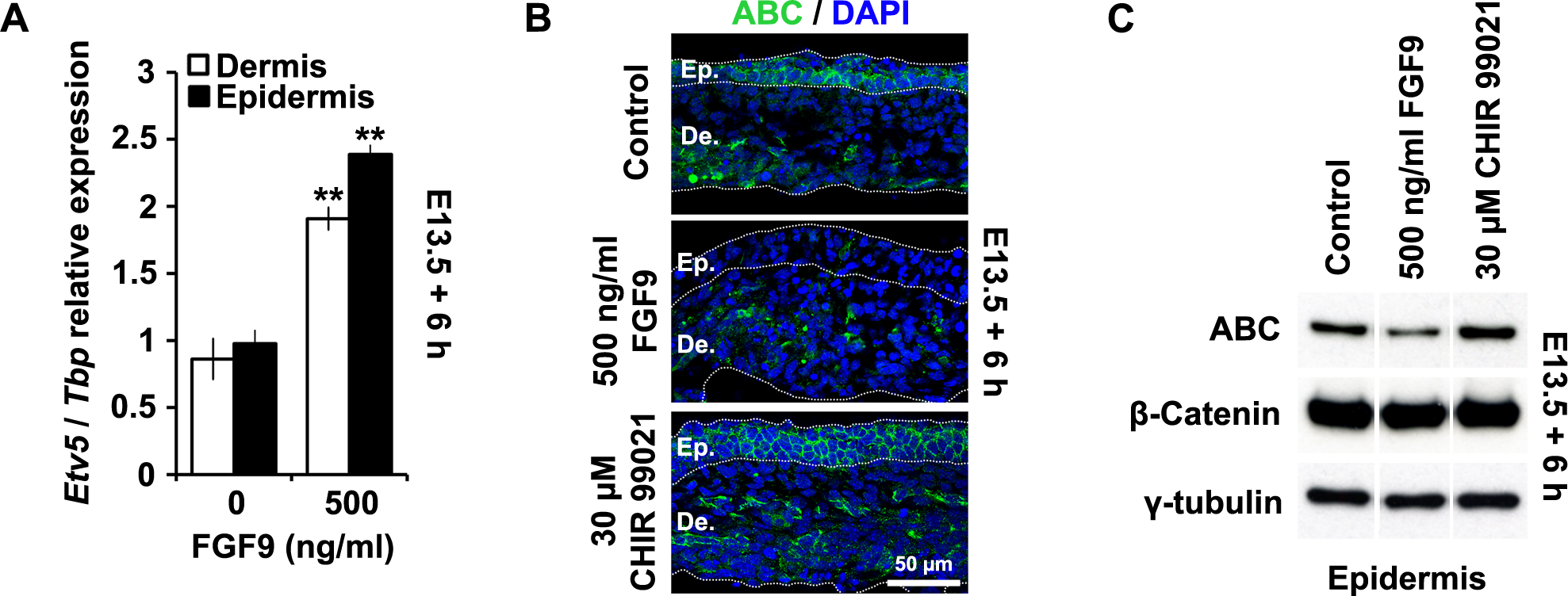

Supplement: S4 Fig — (A) qRT-PCR detecting Etv5 expression in epidermal and dermal fractions of skin cultured with FGF9 for 6 hrs. Gene expression is induced in both tissue compartments. Error bars represent s.e.m. of three independent skins. Statistical significance from control was calculated using a Student t test, (**p < 0.01). The raw numerical values can be found in S1 Data. (B) Immunodetection of active β-catenin (ABC) in cryosections from dorsal skin explants treated with FGF9 or the β -catenin activator CHIR99021 for 6 hrs. Tissue layers are demarcated by dotted lines. Ep. = Epidermis, De. = Dermis. Scale bar as annotated. (C) Immunoblot determination of ABC in isolated epidermis of skin explants treated with FGF9 or CHIR99021 for 6 hrs. Total β-catenin and γ-tubulin were used as loading controls. (TIF) [file pbio.2002117.s004.tif]

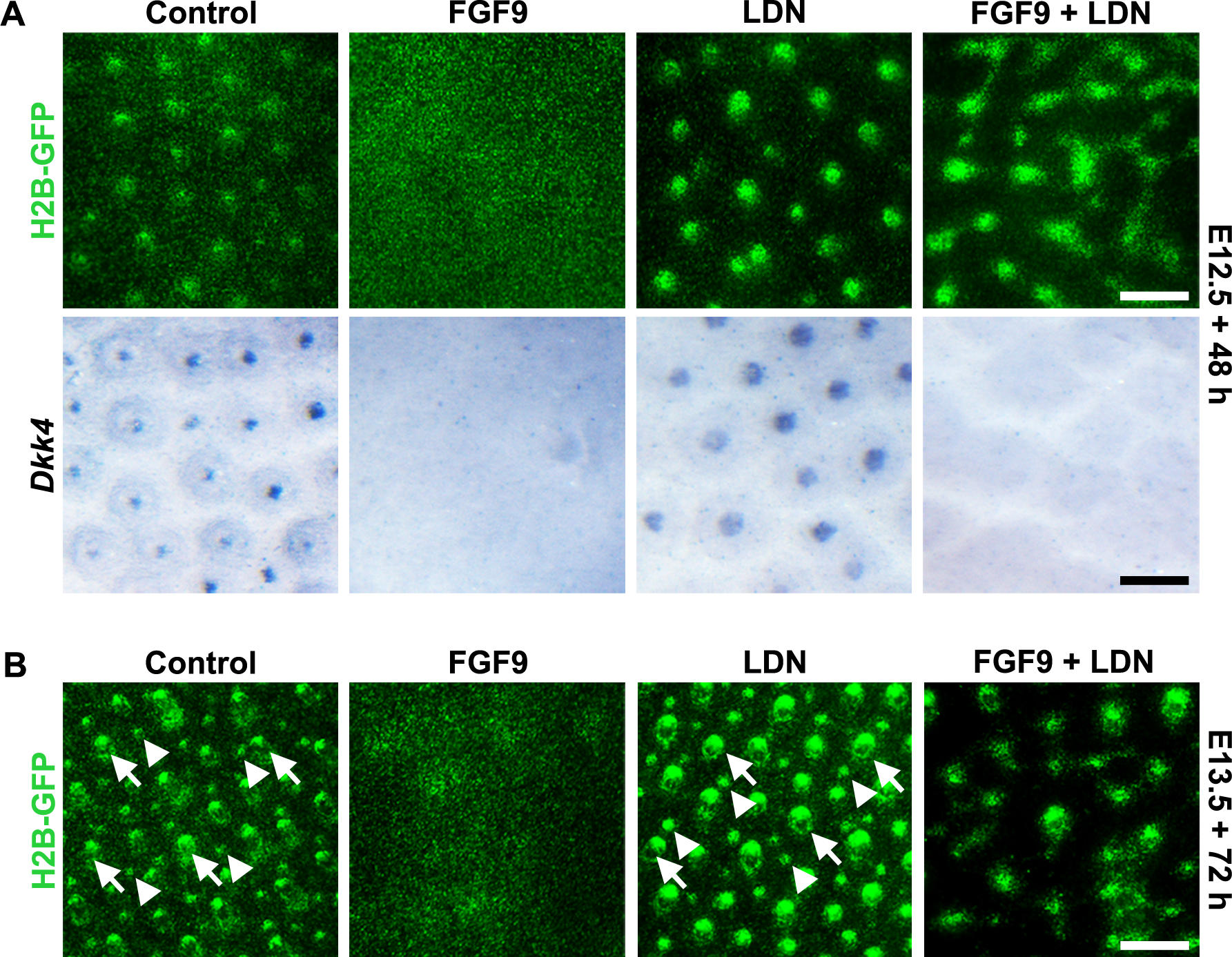

Supplement: S5 Fig — (A) E12.5 TCF/Lef::H2B-GFP dorsal skin explants cultured with either FGF9 (1 μg/ml), LDN193189 (LDN) (10 μM) or both for 48 hrs show a periodic pattern of cellular aggregates and lack Dkk4 foci. Scale bar: 250 μm. (B) GFP signal in E13.5 TCF/Lef::H2B-GFP dorsal skin explants cultured in FGF9 and LDN for 72 hrs. Arrows indicate primary hair follicle primordia, arrowheads show secondary follicle primordia. Application of FGF and suppression of BMP signalling permits the formation of mesenchymal aggregate patterns at each stage, and secondary follicle primordia are not inserted independently between mesenchymal patterns in FGFHiBMPLo conditions. Scale bar: 250 μm. (TIF) [file pbio.2002117.s005.tif]

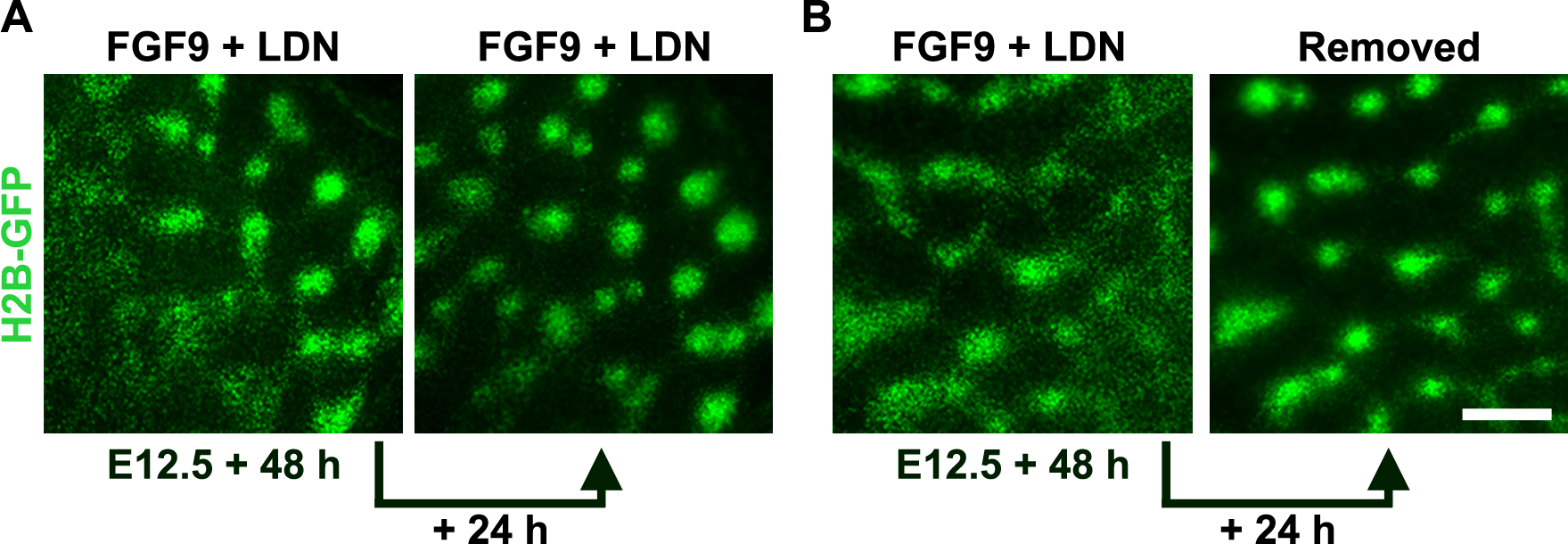

Supplement: S6 Fig — E12.5 TCF/Lef::H2B-GFP skin explants were cultured in the presence of FGF9 (1 μg/ml), LDN193189 (LDN) (10 μM) or both for 27 hours, then imaged and this condition was either reapplied (A) or removed (B) and culture continued for a further 24 hours. Mesenchymal condensations formed in the FGFHiBMPLo conditions are stable and continue to resolve after removal of FGF9 and LDN. Scale bar: 250 μm. (TIF) [file pbio.2002117.s006.tif]

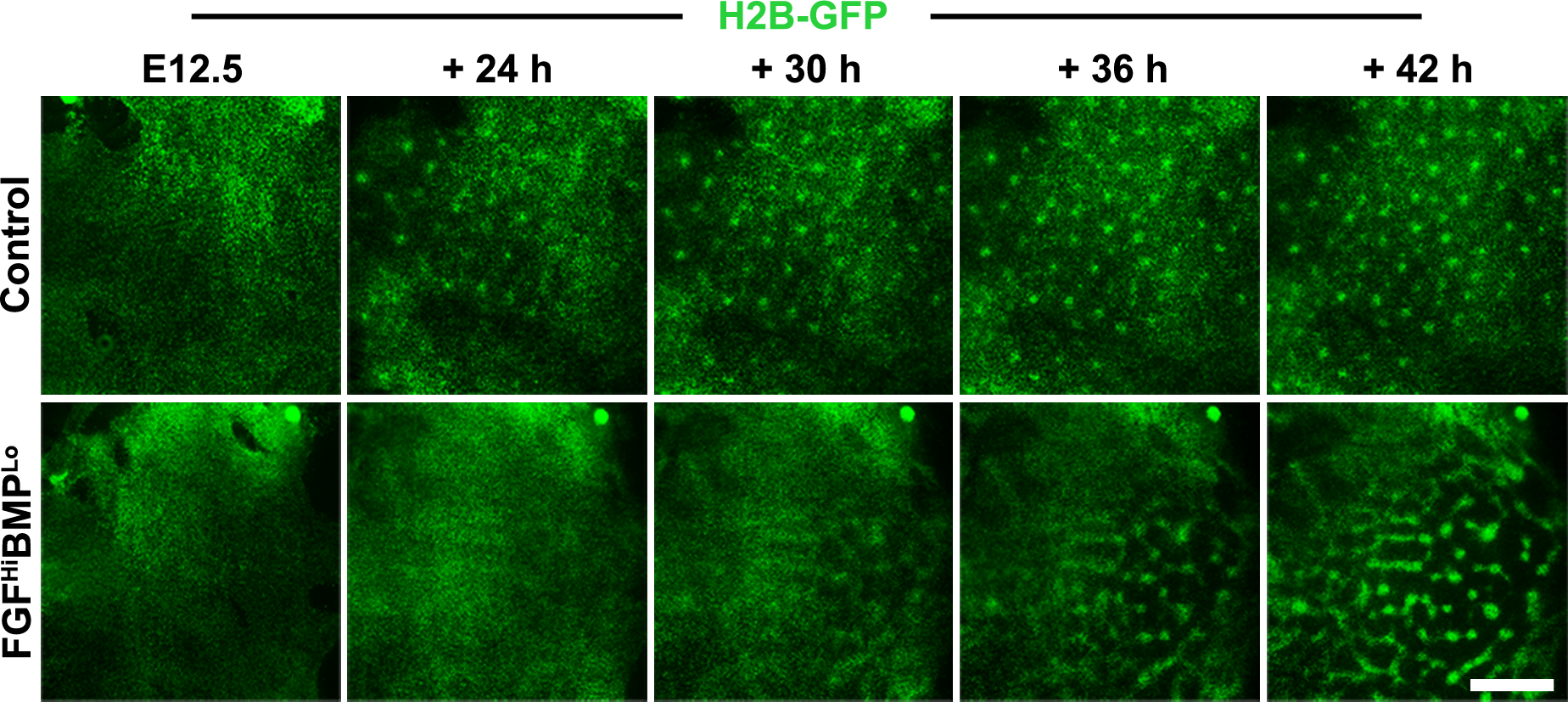

Supplement: S7 Fig — E12.5 TCF/Lef::H2B-GFP skin explants were cultured over a period of 48 hrs and imaged at time intervals as labelled. FGFHiBMPLo pattern formation is slower to resolve than control condensate formation. Scale bar: 1 mm. (TIF) [file pbio.2002117.s007.tif]

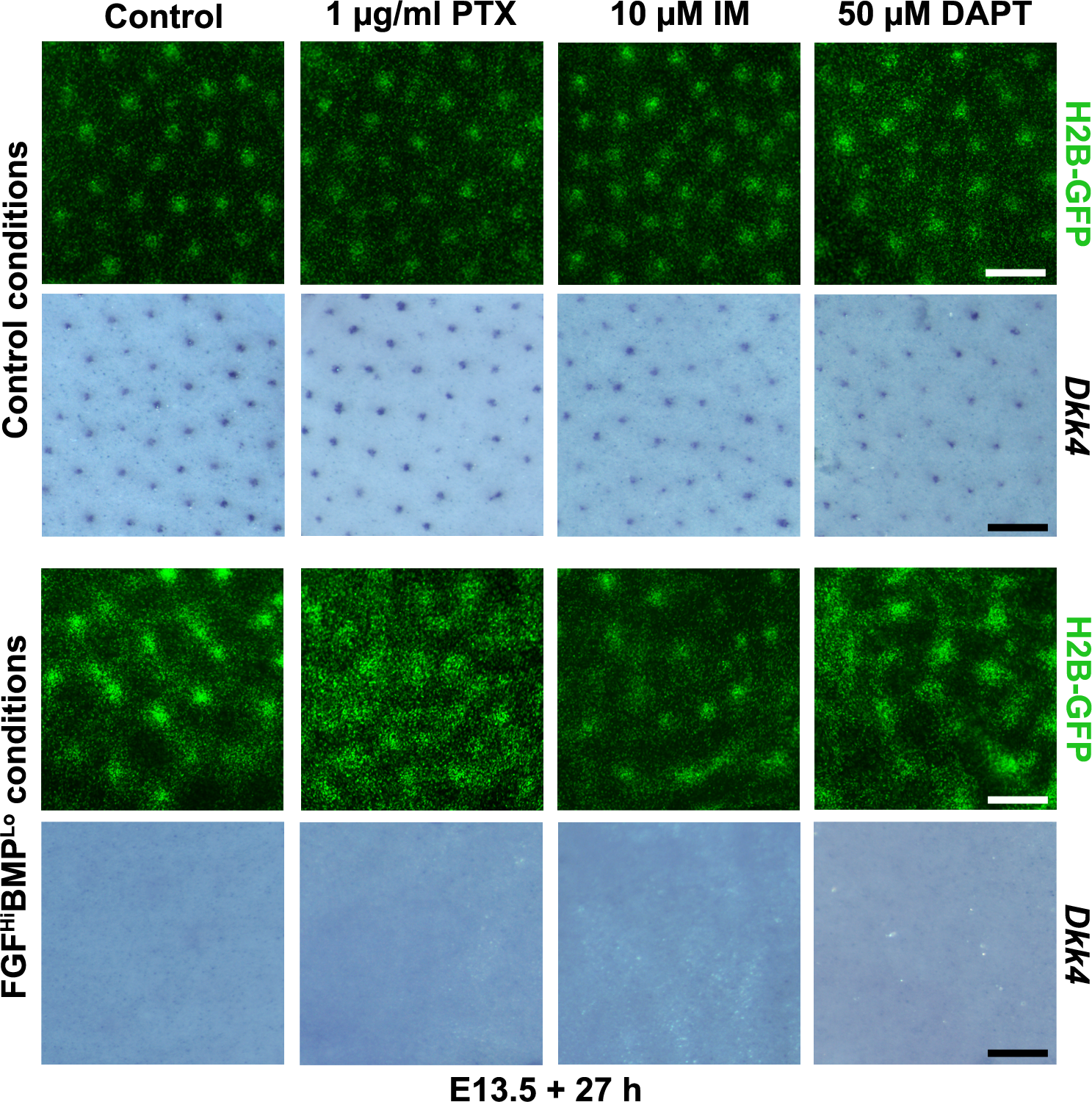

Supplement: S8 Fig — FGFHiBMPLo condensations are formed in the presence of pertussis toxin (PTX, a G-protein inhibitor), imatanib mesylate (IM, a PDGFR inhibitor) and DAPT (a NOTCH inhibitor). Scale bars: 250 μm. (TIF) [file pbio.2002117.s008.tif]

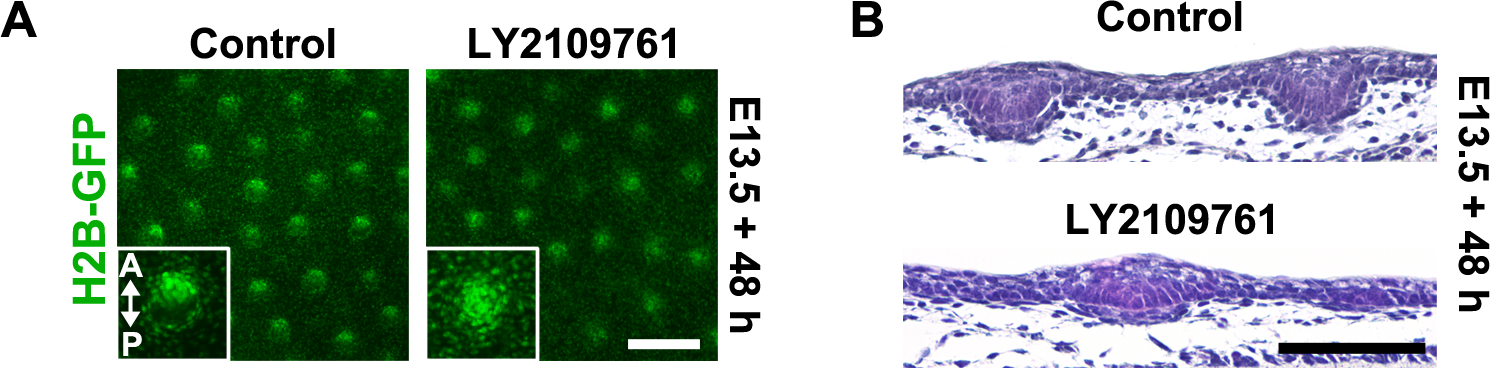

Supplement: S9 Fig — (A) Images of E13.5 TCF/Lef::H2B-GFP skin cultured for 48 hours with or without TGFβ inhibition by LY2109761 (25 μM). Hair follicle primordia in control skin have formed a dermal papilla and polarised from anterior to posterior (inset), while TGFβ inhibited samples have not progressed to this stage. Scale bar: 250 μm. (B) Haematoxylin stained sections of skin samples in panel (A). Placode downgrowth is delayed in the LY2109761 condition, consistent with the reported phenotype of the Tgfb2 mutant. Scale bar: 100 μm. (TIF) [file pbio.2002117.s009.tif]
