# Supplementary material for: Hierarchical patterning modes orchestrate hair follicle morphogenesis
Source: PLoS Biol. 2017 Jul 11;15(7):e2002117. doi: 10.1371/journal.pbio.2002117 (PMC5507405; doi:10.1371/journal.pbio.2002117)
Supplement: S1 Appendix — (DOCX) [file pbio.2002117.s018.docx]

**APPENDIX**

1. **Derivation of gene regulatory network**

**Genome wide mRNA half-life determination and regulatory network derivation**

E13.5 unpatterned dorsal skin explants were treated with Actinomycin D, an inhibitor of RNA synthesis, for 30, 60 or 120 minutes before separation of the epidermal and dermal components, followed by RNA isolation, pooling and RNA sequencing (S3A Fig). We confirmed complete suppression of transcription at the dose of Actinomycin D used by ^3^H-cytidine incorporation assay (S3B Fig). The definitive placode marker *Shh* [[1](#_ENREF_1)] was not detected above threshold levels in our RNA sequencing (RNA-seq) data, verifying that skin explants used did not carry hair placodes at the time of collection. Raw reads obtained from RNA-seq were normalised to *Capzb* abundance. Changes in transcript abundances at the different time points from the normalised RNA-seq data set were used to calculate half-lives for all mRNAs expressed in the epidermis and dermis separately (S2 Table). From RNA-seq data, transcripts from ~12,000 genes were detected over threshold levels in the epidermis and ~12,500 were detected in the dermis. ~9% of all mRNAs detected in the epidermis, and ~16% of those in the dermis, presented with half-lives of 90 minutes or less (S3C Fig). As a validation, we assessed the half-lives of a selected group of transcripts in independently treated embryonic skin cultures by qRT-PCR, which yielded results similar to those obtained by RNA-seq (S3D and S3E Fig).

We next filtered the short-lived transcripts (t_1/2_ ≤90 minutes) to focus only upon extracellular molecules (ligands, receptors and extracellular signal-attenuating molecules) in the BMP, FGF and WNT pathways (S3 Table).

**i) Candidate determination**

Candidates were selected from a list of diffusible ligands, receptors and extracellular signal-attenuating molecules involved in the BMP, FGF and WNT pathways. This list was compiled from lists of the transcripts detected by commercially available pathway-specific PCR-arrays. Transcripts with epidermal or dermal mRNA half-lives of 90 minutes or less, as measured by RNA-seq, were designated as candidates. Transcripts with a mean t_0_ corrected transcript abundance value of less than 0.001, or mean raw reads of less than 60, were excluded from candidate selection.

**ii) Determination of regulatory relationships**

E13.5 dorsal skin explants were treated for 6 hours with small molecules or recombinant proteins to either stimulate or repress BMP, FGF and WNT signalling pathways. The response of all candidate genes to each treatment was assessed by qRT-PCR (normalising to *Tbp*). A candidate was judged to be responsive if it showed minimum 1.8 fold, statistically significant (p≤0.05) increase or decrease in expression in response to treatments with opposing effects on a pathway. If a candidate showed a response to pathway stimulation, but not repression, the candidate was classed as regulated. If a candidate transcript responded in the same direction to both stimulation and repression of a given pathway, or responded only to repression of the pathway, that candidate was classed as not regulated by that pathway.

**iii) Network reduction**

To build the 5-species network, candidates were grouped into WNT, DKK (WNT inhibitors), FGF, BMP and BMP-inhibitors species groups. If a candidate within a species group was found to be regulated, that interaction was applied to the species as a whole (e.g., negative regulation of *Fgf20* by BMP signalling was interpreted as BMP inhibition of FGF) and represented by a + or – sign on the matrix. When different members of the same species group were regulated in opposite directions by the same pathway, the overall impact on the species was assessed by normalising fold changes to mean t_0_ expression level and calculating net upregulation or downregulation of all species.

**B. Mathematical analysis of reaction-diffusion network: Background**

The molecular signalling network is mathematically represented by a system of reaction-diffusion equations, where each equation describes the spatiotemporal change in the concentration of a signalling molecule. For example, if $n_{F}(x,t)$ describes the concentration of FGF at time $t$ and position $x$, then

$$\frac{\partial n_{F}}{\partial t}=f_{F}\left( n_{F},n_{W},n_{D},n_{B},n_{Bi} \right)+D_{F}\Delta n_{F}.$$

The first term on the right-hand-side (represented by function $f_{F}$) defines the kinetics and describes the rate of change of $n_{FGF}$ due to, e.g. upregulation, downregulation, background degradation. This will depend on both its own concentration as well as those of other signalling components (*F=*FGF*, W=*WNT*, D*=DKK, *B=*BMP*, Bi=*BMP inhibitors). The second term describes diffusion (where the operator $\Delta$ denotes the Laplacian) and is parametrised by a nonnegative diffusion coefficient $D_{F}.$Note that $D_{i}=0$ for non-diffusing entities (e.g. transmembrane receptors). Equivalent equations can be written for each of the other signalling components in the network and the result is a system of equations which can be written in vector form

$$\frac{\partial n}{\partial t}=f\left( n \right)+D\Delta n, (1)$$

where ${n=(n}_{F},n_{W},n_{D},n_{B},n_{Bi})$ is a column vector of concentrations, ${f=(f}_{F},f_{W},f_{D},f_{B},f_{Bi})$ is a column vector describing the kinetic terms and $D$ is a diffusion matrix with diffusion coefficients of corresponding species on its diagonal.

A typical first step would be to postulate specific nonlinear functional forms for the various kinetic terms, according to the known interactions in the network: for example, the positive effect of WNT on FGF can be included through ensuring that $f_{F}$ increases with $n_{W}$. Our fundamental objective, however, is to test whether the signalling network can generate a spatially periodic pattern and so we proceed directly to a linear stability analysis, where we search whether the conditions for a Turing instability (TI) are met according to the work of Turing [[2](#_ENREF_2)]. For a homogeneous (or uniform) steady state (representing the initial nonpatterned tissue) of equations (1), a TI arises if (i) it is stable in the absence of diffusion terms, and (ii) it is driven unstable through the addition of diffusion terms. Under this scenario, the nonpatterned tissue can self-organise into a spatial pattern of varying signalling levels, hence providing a template for tissue differentiation and morphogenesis. We use the same approach employed by Turing in his seminal paper to investigate these requirements.

Within this investigation of linear stability we can effectively describe the dynamics of system (1) in terms of a system of linear partial differential equations, in matrix form

$$\frac{\partial n}{\partial t}=Jn+D\Delta n$$

where $J$ is a matrix of constant coefficients obtained through linearizing the kinetics about the uniform steady state. We circumvent the need to formally define the original functions $f$ from which $J$ is defined by noting that the sign of $J_{ij}$ describes the positive or negative influence of species $n_{j}$ on the time evolution of species $n_{i}$: i.e. upregulation of $n_{i}$ by $n_{j}$ will be represented by a positive value for $J_{ij}$. Consequently, $J$ can be informed directly from the sign structure of the experimentally identified matrix in S3F Fig (specifically, its transpose $J^{T}$ follows the same sign structure as S3F Fig).

Turing instability (diffusion-driven instability) requires stability of the zero steady state without diffusion (stability with respect to spatially homogeneous perturbations) and instability once diffusion is present (instability with respect to spatially inhomogeneous perturbations). As some species are not diffusing (e.g. transmembrane receptors) additional constraints on kinetics have to be satisfied, particularly the reduced kinetics of the non-diffusing species must be stable on its own [[3](#_ENREF_3)].

Stability can be readily assessed via the Routh-Hurwitz conditions (guaranteeing that all eigenvalues lie in the left half-plane of complex numbers) and instability via inspection of the roots of a quintic polynomial, the so-called dispersion relation [[4](#_ENREF_4)]

$$Det\left( J-\lambda I-k^{2}D \right)$$

where the parameter $k$ relates to an eigenfunction $\gamma_{k}$ through a coupled spatial problem

$-\Delta\gamma_{k}=k^{2}\gamma_{k}$, subjected to appropriate boundary conditions (e.g. $k^{2}=\left( \frac{m\pi}{L} \right)^{2}$for zero-flux boundary conditions on a one dimensional domain (0,L)). Linear stability occurs when the real parts of all eigenvalues are negative, i.e. $\mathfrak{R}\left( \lambda\left( k^{2} \right) \right)<0$ where the eigenvalues $\lambda\left( k^{2} \right)$ are the rootsof the above dispersion relation, with instability occurring if this condition is not met (this follows from the fact that $\lambda$ is the exponential growth rate constant of morphogen concentration, i.e. $n\left( t,x \right)=n_{0}e^{\lambda t}g\left( x \right)$). We shall consider a 1D domain for simplicity as the existence of pattern on 2D rectangular domain can be straightforwardly assessed from the 1D case. We assume Neumann boundary conditions (zero flux), meaning that morphogens are trapped within the considered tissue specimen. Additionally, we focus on non-temporal pattern formation because, in the context of pattern formation, Hopf bifurcations are not particularly relevant as oscillations are not typically observed in putative Turing pairs (e.g. [[5](#_ENREF_5)]).

**C. Mathematical analysis of reaction-diffusion network: assessment of Turing Instability in specific cases**

We shall use the following notation in indices for referring to diffusion and matrix components: F for FGF, W for WNT, D for DKK, B for BMP, Bi for BMP inhibitors.

In order to have Turing instability (TI), the dispersion curve must intersect with the$k=0$ axis below zero (the condition $\mathfrak{R}\left( \lambda(0) \right)<0$ is equivalent to the requirement for stability in the absence of diffusion) and again must drop below $\mathfrak{R}\left( \lambda\right)=0$ axis for large $k^{2}$ (so that the continuous description does not break down and a critical domain size exists, see [[3](#_ENREF_3)]). Further, the dispersion curve must cross the real axis into the upper half-plane (equivalent to linear instability, see e.g. [[4](#_ENREF_4)]) and, as we require a time-independent pattern $\mathfrak{R}\left( \lambda\right)=\lambda$ (a non-zero imaginary component, $\mathfrak{I}\left( \lambda\right)$, would generate time oscillations of the pattern), the root of dispersion relation must be directly zero at the critical instability. Thus the coefficient by $\lambda^{0}$ (the so-called absolute term) must vanish.

Note that we treat cases where no response was observed as strictly without any response at all to simplify the analysis. However, as we aim for a model for a spatial self-organisation in biology, robustness is required. Hence we expect that a faint response can be represented by strict zero in the linearised kinetics. As we are investigating whether the identified network is a plausible one for pattern formation as a proof of concept, we regard these simplifications as reasonable.

As there is a strict distinction in conditions for Turing instability in networks with diffusing and non-diffusing morphogens [[3](#_ENREF_3)] we distinguish several cases for the network shown in Fig 1E:

1. **Non-diffusing WNT and non-diffusing BMP inhibitors.** As both $J_{WBi}=0=J_{BiW}$ , the absolute term in the dispersion relation is a quadratic polynomial and hence, in order to meet the above conditions for TI, it is required to have two positive roots. A quadratic polynomial $k^{2}+b_{1}k+b_{2}=0$ has two positive roots if$b_{1}<0$ and $b_{2}>0$. Since $b_{1}={(D}_{F}J_{BBi}J_{DW}J_{BiB}J_{WD}-D_{D}J_{BBi}J_{BiB}J_{FF}J_{WW})/D_{F}D_{D}J_{BBi}J_{BiB}J_{WW}$ the condition $b_{1}<0$ is in contradiction with the identified network in Fig 1E as both terms are positive.
2. **Non-diffusing BMP inhibitors only.** The absolute term is a cubic polynomial in *k_­_^2^* and thus to meet the above conditions for TI, it is required to have three real roots, one negative and two positive. The roots are

$$k^{2}=\frac{J_{FF}}{D_{F}},k^{2}=\frac{J_{WW}}{2D_{W}}\pm\frac{1}{2D_{W}}\sqrt{4\frac{D_{W}}{D_{D}}J_{WD}J_{DW}+J_{WW}^{2}}$$

and hence TI cannot occur (namely as $J_{WW}<0$, $J_{WD}J_{DW}<0$ , and $J_{FF}<0$).

1. **Non-diffusing WNT molecules only.** This network shows TI. Algebraic conditions can be readily obtained as outlined above but they allow a very limited insight. An example that gives a rise to pattern is

$$D_{F}=0.02,D_{D}=0.66,D_{B}=0.008,D_{Bi}=0.53$$

$$J=\left( \begin{matrix} -0.01 & 0.25 & 0 & -0.27 & 0 \\ 0 & -0.6 & -0.2 & -0.45 & 0 \\ 0 & 0.46 & 0 & -0.29 & 0 \\ -0.24 & 0.66 & 0 & -0.29 & -0.41 \\ 0 & 0 & 0 & 0.47 & 0 \end{matrix} \right)$$

and the corresponding dispersion curve is the following:


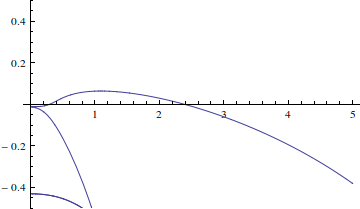


1. **All species diffusing.** This network shows TI. Algebraic conditions can be readily obtained as outlined above but they allow a very limited insight. An example that gives a rise to pattern is

$$D_{F}=0.03,D_{W}=0.38,D_{D}=0.29,D_{B}=0.038,D_{Bi}=0.84$$

$$J=\left( \begin{matrix} -0.14 & 0.41 & 0 & -0.83 & 0 \\ 0 & -0.48 & -0.38 & -0.48 & 0 \\ 0 & 0.07 & 0 & -0.25 & 0 \\ -0.36 & 0.92 & 0 & -0.78 & -0.78 \\ 0 & 0 & 0 & 0.85 & 0 \end{matrix} \right)$$

and the corresponding dispersion curve is the following:


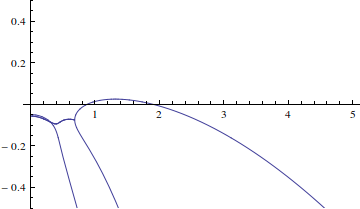


**D. Particle image analysis**

Particle image analysis (PIV) was conducted using MatPIV [[6](#_ENREF_6)]. PIV detects cell displacement by correlation analysis of small image segments [[7](#_ENREF_7)]. Hereby, the cross-correlation between small interrogation areas of frame *i,* centred at position *(x,y)* with interrogation areas of frame *i+1,* centred at *(*$x\pm n,y\pm m$*)* for *n,m* $\in$ *[0,N],* are computed*.* A two-dimensional Gaussian distribution is fitted to the cross-correlation matrix. The peak of the Gaussian is interpolated and provides the average displacement of the cells positioned around *(x,y)* with subpixel resolution. The size of the image segments has to be adjusted to the average cell size to yield reliable results. For our data we used 32x32 pixel windows, overlapping by 50%. Spurious displacement vectors were removed by applying a global, local and signal-to-noise-ratio filter. When applied to every pair of consecutive frames, PIV yields a displacement field for every recorded time point, and thus spatio-temporal information on cell velocities.

PIV-derived pseudo-trajectories (pathlines) represent average paths of (groups of) cells initially located in a given region. Hereby 3000 randomly chosen positions were subjected to the displacement field for the given time period.

**E. Simulations of edge effects on pattern behaviour**

**Mathematical model.**

We numerically solve a cell-chemotaxis model. The model is governed by two variables: $n(x,y,t)$ and $c(x,y,t)$, where $\left( x,y \right)\in\left[ 0,L \right]\times\left[ 0,L \right]$ denotes position in a two-dimensional square region of tissue and t $\in\left[ 0,\infty\right)$ denotes time. We note that for convenience the model is presented in a standard “non-dimensional” form, where variables have been scaled to unitless quantities in a manner that reduces the number of free parameters.

The cell-chemotaxis model follows a classic Keller-Segel type [[8](#_ENREF_8)]. Setting $n\left( x,y,t \right)$ to denote the density of mesenchymal cells and $c\left( x,y,t \right)$ as its corresponding chemoattractant, the equations are given by:

$$\frac{\partial n}{\partial t}=D\nabla^{2}n-\chi\nabla\cdot\left( n(1-n/k)\nabla c \right);$$

$$\frac{\partial c}{\partial t}=\nabla^{2}c+n-c.$$

In the equation for $n$ the first equation on the right hand side describes random motility, while the second term describes chemotactic movement of cells up gradients of chemoattractant $c$. The $(1-n/k)$ factor describes a “volume-filling” effect that limits excessive accumulation of cells, for example due to tissue capacity [[9](#_ENREF_9)]. The equation for $c$ includes terms for diffusion, production of the attractant by the cells and decay/degradation. We note that it is assumed that cell growth is negligible over the time scale of simulations.

**Parameters and Initial Conditions**

Standard linear stability analysis (e.g. [[10](#_ENREF_10)]) shows that under suitable parameter values the above model is capable of self-organisation/pattern formation, in which spatial patterns emerge from noise from an otherwise spatially uniform initial condition: taking the form of spatially separated aggregations of high cell density. Parameters employed in the simulations ($D=0.01, k=20, \chi=2,L=15$) are chosen to ensure that this pattern formation outcome can occur. Initially we set any cell densities at a spatially constant level and chemical concentrations at their uniform steady state values, with chemicals also perturbed by a small random component to represent environmental noise. Specifically, we set:

$$n\left( x,y,0 \right)= 1 \mathrm{and} c\left( x,y,0 \right)= 1+\boldsymbol{r}\left( x,y \right)$$

In the above, the random component $\boldsymbol{r}\left( x,y \right)$ is uniformly chosen from [-0.01,0.01], effectively describing an up to 1% perturbation from the steady state value. Note that the initial cell density in the above has been scaled to unity as a result of the previously mentioned nondimensionalisation.

**Boundary conditions**

Our region describes a square region of tissue where one side follows along a tissue edge, see schematic below. The internal boundaries $\Gamma_{A},\Gamma_{B},\Gamma_{C}$ refer to “imaginary” lines within the tissue and no special boundary conditions are required. Here we chose a sufficiently large spatial region and minimise any impact from the boundary conditions along these edges via the standard choice of zero-flux conditions for cells and chemicals as follows:

$\boldsymbol{n}\cdot(D\nabla n-\chi n(1-n/k)\nabla c$) = 0, $\boldsymbol{n}\cdot\nabla c=0$ on $\Gamma_{A},\Gamma_{B},\Gamma_{C}$.

Note that the vector $\boldsymbol{n}$ denotes the outer unit norms along boundaries $\Gamma_{A},\Gamma_{B},\Gamma_{C}$.

The tissue edge boundary $\Gamma_{D}$ demands closer inspection. We assume cells are unable to move across the boundary (e.g. due to the lack of a suitable substrate/environment outside the tissue) and therefore specify zero-flux boundary conditions for the cell population. For the chemical it is assumed that this can freely diffuse across the tissue edge and into the extra-tissue region, where it is assumed no chemical synthesis can occur (i.e. no cells exist to synthesise the chemical). Consequently, in the extra-tissue region the concentration of chemical substance ($c_{ext})$ would simply be governed by diffusion and decay:

$$\frac{\partial c_{ext}}{\partial t}=\nabla^{2}c_{ext}-c_{ext}.$$

Following a standard approach (e.g. see [[11](#_ENREF_11)]) the above is solved and, following appropriate assumptions of continuity at the tissue edge, we arrive at the following Robin condition along $\Gamma_{D}$ for the tissue chemical concentration:

$$\mathbf{n}.\nabla c=-\mu c.$$

Effectively, the above describes a “lossy” boundary in which chemical flows off of the tissue edge, where it simply diffuses and decays. Note that the loss rate $\mu$ will be determined by the rates of diffusion and decay, and under their scaling to unity we have $\mu=1$. Summarising, we have the following boundary conditions on $\Gamma_{D}$:

$\boldsymbol{n}\cdot(D\nabla n-\chi n(1-n/k)\nabla c$) = 0, $\boldsymbol{n}\cdot\nabla c=-\mu c$ on $\Gamma_{D}.$

**Numerical Simulations**

The model is numerically solved using a standard Method of Lines approach. The spatial region is discretised into a uniform square lattice of inter-lattice spacing $\Delta x$. Diffusion terms are solved using a central order difference scheme, while advection terms (the chemotaxis operator) employ a third-order upwind scheme with flux limiting to preserve positivity. The resulting system of equations is integrated in time using a stiff-system integrator (ROWMAP). In the numerical simulations we use a discretisation spacing $\Delta x=0.05$ and set absolute/relative tolerances in ROWMAP at ${10}^{-8}.$ Numerical simulations have been cross-validated via testing at different discretisations and tolerances.


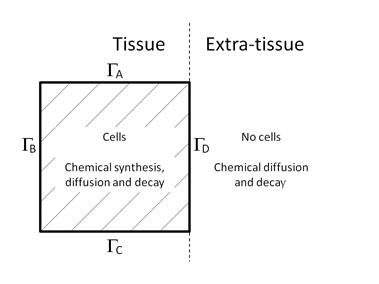


Schematic showing the computational domain. This defines a square region of tissue with one boundary representing a tissue edge.

**References for Appendix**

1. Levy V, Lindon C, Harfe BD, Morgan BA (2005) Distinct stem cell populations regenerate the follicle and interfollicular epidermis. Dev Cell 9: 855-861.

2. Turing AM (1952) The Chemical Basis of Morphogenesis. Philosophical Transactions of the Royal Society of London Series B-Biological Sciences 237: 37-72.

3. Klika V, Baker RE, Headon D, Gaffney EA (2012) The influence of receptor-mediated interactions on reaction-diffusion mechanisms of cellular self-organisation. Bull Math Biol 74: 935-957.

4. Murray JD (2002) Mathematical Biology I: An introduction. New York, NY, USA: Springer.

5. Schier AF (2003) Nodal signaling in vertebrate development. Annu Rev Cell Dev Biol 19: 589-621.

6. Sveen JK (2004) An introduction to MatPIV v.1.6.1. Mechanics and Applied Mathematics. Oslo: Dept of Math, University of Oslo.

7. Raffel M, Willert C.E., Wereley S., Kompenhans J. (1998) Particle Image Velocimetry - A Practical Guide. Berlin Heidelberg: Springer-Verlag.

8. Keller EF, Segel LA (1970) Initiation of slime mold aggregation viewed as an instability. Journal of Theoretical Biology 26: 399-415.

9. Hillen T, Painter KJ (2009) A user's guide to PDE models for chemotaxis. J Math Biol 58: 183-217.

10. Murray JD (2003) Mathematical biology II: spatial models and biomedical applications.: Springer.

11. Bianchi A, Painter KJ, Sherratt JA (2016) Spatio-temporal Models of Lymphangiogenesis in Wound Healing. Bull Math Biol 78: 1904-1941.
