# Supplementary material for: Hierarchical patterning modes orchestrate hair follicle morphogenesis
Source: PLoS Biol. 2017 Jul 11;15(7):e2002117. doi: 10.1371/journal.pbio.2002117 (PMC5507405; doi:10.1371/journal.pbio.2002117)
Supplement: S1 Supporting Methods — (DOCX) [file pbio.2002117.s019.docx]

**Supporting Methods**

**Whole skin time-lapse imaging**

For the FGF9/BSA bead and the FGF9 and LDN193189 culture experiments, real time imaging was performed using the Zeiss Live Cell Observer/Deconvolution system with an incubated CO_2_ stage using Zen 2012 software (Zeiss).


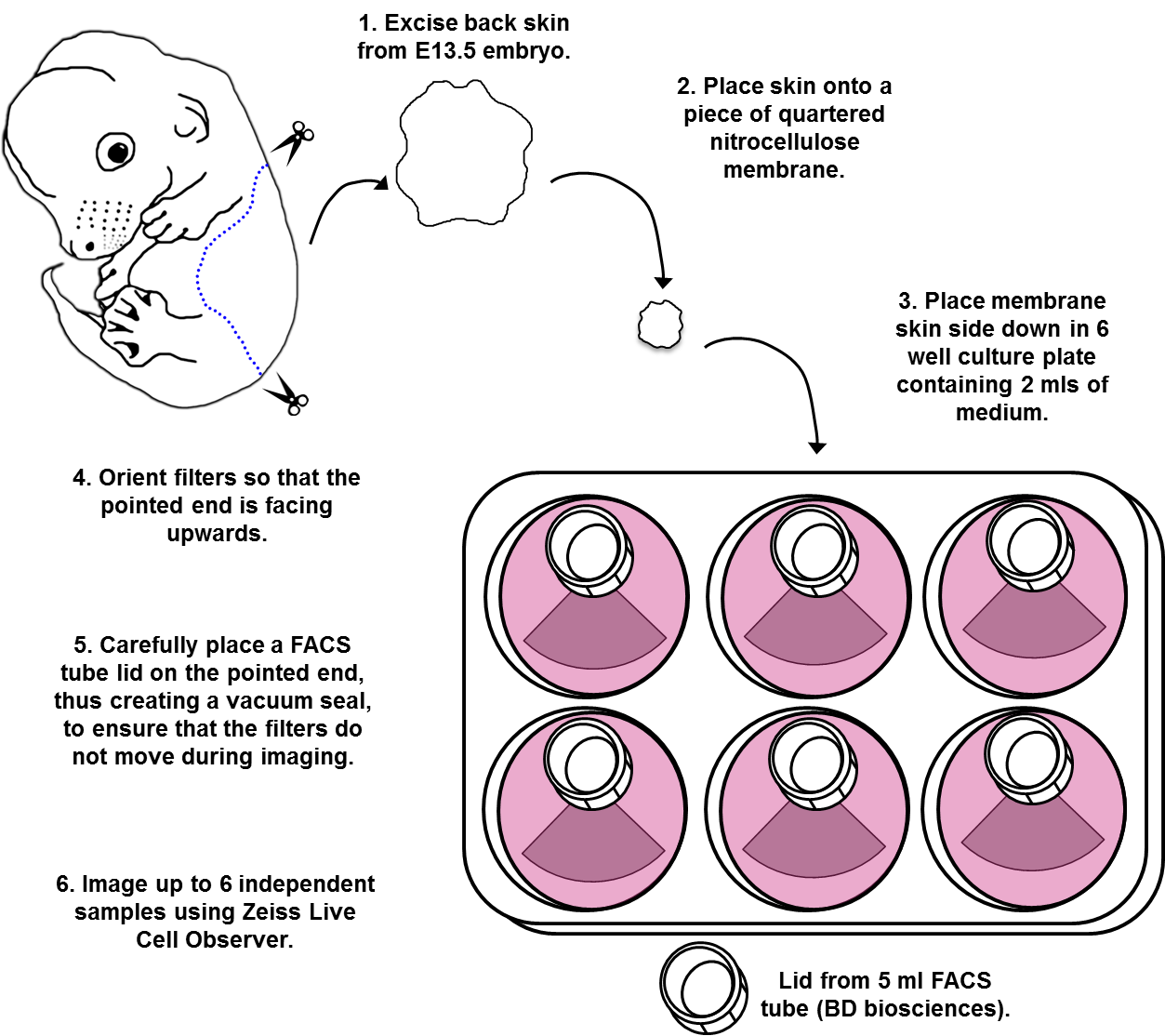


**Figure A. Schematic of experimental set up for real time imaging experiments.**

The incubation stage was maintained at 37°C and with a 5% CO_2_ atmosphere throughout the course of the experiment. TCF/Lef::H2B-GFP dorsal skin explants, prepared as previously described, were placed epidermal side down into individual wells of a 6-well dish containing 2 ml of complete DMEM and supplemented with recombinant proteins/small molecule inhibitors according to experiment. Milli-Q water was placed into empty wells or spaces between the well to maintain humidity. The plate was placed onto the microscope stage and skins manoeuvred with a pipette tip. A sterile plastic weight (FACS tube lid) was placed at the tip of the nitrocellulose filter paper, away from the skin. Images were captured using a 5X or 10X objective at 10 minute intervals over a period of 48 to 72 hours.

**Oligonucleotide sequences used for qRT-PCR**

| **Transcript** | **Forward / Reverse oligonucleotides** |
| --- | --- |
| *Axin2* | ctctccttccagatcccagc / ctggaccttgcccacactag |
| *Bambi* | atcgccactccagctacttc / tgagcagcatcacagtagca |
| *Bmp2* | cggactgcggtctcctaa / ggggaagcagcaacactaga |
| *Bmp4* | gaggagtttccatcacgaaga / gctctgccgaggagatca |
| *Capzb* | aagttgacctccacggtgat / acagtttcgtctttctccatctg |
| *Col1A1* | tggacctccggctcctgctc / acgtctcaccattggggaccct |
| *Ctgf* | gcagctgggagaactgtgta / acagggtgcaccatctttg |
| *Ctnnb1* | ttggatatcgccaggatgat / tcaactggatagtcagcacca |
| *Dkk1* | ccgggaactactgcaaaaat / ccaaggttttcaatgatgctt |
| *Dkk4* | gcctatgcagaaggaaccac / gacagctttctccctcctgtc |
| *Edar* | tgtcctccatgcagaccag / gcatatctgataacctcctttgg |
| *Etv5* | tagcggagactttggaagca / cctgggaccataaaagggac |
| *Fgf7* | aacgaggcaaagtgaaaggga / attgccacaattccaactgcc |
| *Fgf9* | ggactcttacctcggcatgaa / tgtaccagttctcttcaaactgt |
| *Fgf10* | cgggaccaagaatgaagact / aacaactccgatttccactga |
| *Fgf18* | agtcaagtccggatcaaggg / tctcaatgaacacgcactcc |
| *Fgf20* | gtggacagtggcctgtacct / gacccgtgtctccatgttta |
| *Fn* | ctcatcagcatccagcag / gggagcaggtcaggaatg |
| *Fzd1* | ccaccttccagctttattagagg / tacagggaggctgggtca |
| *Fzd2* | gtctccgaccctcagtccga / agagttggggagattgggcg |
| *Fzd4* | caactttcacgccgctcatc / tgcacattggcacataaaccg |
| *Fzd7* | gcttcggattccgtgcaac / atggctcgttctcgctttga |
| *Fzd8* | tgttggaagtgacctcgctc / aacggcaccgtgatctcttg |
| *Fzd10* | tgctgcctgtgcataaactt / cccccaggaaagctctttag |
| *Krt5* | ctacaggaagctgctggagggc / aatgctgctgcctcctccgta |
| *Nog* | atccaagtctgtgcacctca / acactcggaaatgatggggt |
| *Rgma* | accaatacacctgtgctgcc / ctggtccacacactcttggaa |
| *Rgmb* | cctttcggttcaagtgacga / actcgtgctgtgctttgaag |
| *Ror1* | aagctgcaaactgcatacgg / ttggtcacactgacggttcc |
| *Ror2* | gaactgtgtgacgtaccccc / aggaagaacaggcaagcgat |
| *Sdc1* | ccttttggacaggaaggaagtg / tccttcttcttcatccggtacag |
| *Smad7* | ccctcctccttactccagatacccaat / atctggacagcctgcagttggttt |
| *Sostdc1* | aacagcaccctgaatcaagc / gtatttggtggaccgcagtt |
| *Tbp* | tgtgcacaggagccaaga / ccccaccatgttctggat |
| *Tgfbi* | gcccttgaaatcttcaaacaggcgtc / tcctctccagtaaccgctgatagaca |
| *Tgfbr3* | gtttgcagcatttgtgatcg / tgctctgagtgctccctatg |
| *Thbs2* | aggtgcatctcgagagagtcact / ctgcaaacacgagatggacattc |
| *Thbs4* | agggtgtcgggatcaactttgcta / acacacgctccattctgacactca |
| *Tnc* | accatgctgagatagatgttccaaa / cttgacagcagaaacaccaatcc |
| *Tsku* | atgctgtgctctctgttcct / ctcttcttcacactggcagc |
| *Wnt5b* | acaccagtttcgacagaggc / tacgtgaaggcagtctctcg |
| *Wnt9a* | ccttcggccgcctacttc / caggccttgtagtgtgcttg |
|  |  |

**Genome wide mRNA half-life determination**

**Actinomycin D dose determination**

To determine the dose of Actinomycin D sufficient to block transcription, E13.5 dorsal skin cultures were co-treated with 50 µM [^3^H]-labelled cytidine (5’–^3^H, American Radiolabelled Chemicals) and graded doses of Actinomycin D for 3 hours. Explants were washed in PBS, RNA was extracted using Tri Reagent then column purified (RNAeasy mini, Qiagen) to remove unincorporated radionucleosides. RNA quality and concentration was determined using a spectrophotometer (Nanodrop), and radioactivity quantified in counts per minute (CPM) in a scintillation counter. CPMs were adjusted to account for background counts, and normalised to each RNA concentration (S3B Fig).

**Actinomycin D treatment**

E13.5 embryos were harvested and staged as described. Embryos younger than E13.5, or those with visible primary hair follicle primordia present, were excluded from the experiment. Immediately prior to culture, each skin was halved along the midline. One half was incubated immediately (t_0_) in Dispase II supplemented with Actimomycin D (50 µg/mL), and epidermis/dermis separation was performed as described. The two tissue layers were placed separately into Tri Reagent for RNA extraction. The remaining portion of skin (t_x_) was cultured in complete DMEM supplemented with Actinomycin D (50 µg/mL) for 30, 60 or 120 minutes. To minimise inter-sample variation within timepoints, all explants treated for a particular time period were from a single litter of embryos. At the end of the culture period, the explant was processed for epidermis/dermis separation as described, and the two tissue layers reserved separately in Tri Reagent for RNA extraction.

**Quality control**

RNA was extracted as described, and tested for yield and purity using a spectrophotometer (Nanodrop). If the 260/280 ratio was below 1.8, that sample and its t_0_/t_x_ pair was excluded from further analysis. cDNA was synthesised from 50 ng RNA, and qRT-PCR performed for the dermal marker *Col1A1* and epidermal marker *Krt5* to assess the accuracy of each epidermal/dermal separation. Individual epidermal samples with a *Col1A1* expression level exceeding 0.5% of the mean *Col1A1* expression level of the littermate dermal samples were excluded from further analyses along with their t_0_/t_x_ pair. Dermal samples exhibiting a *Krt5* expression level of more than 3% of the mean *Krt5* expression level of the littermate epidermal samples were excluded.

**Sample preparation**

Samples which passed quality control were pooled according to treatment time and purified using an RNeasy mini kit (Qiagen), and the RNA integrity of each pool was assessed using an Agilent Bioanalyzer.

**RNA sequencing**

Library preparation and sequencing were carried out at ARK-Genomics (Edinburgh Genomics), The Roslin Institute. mRNA sequencing libraries were constructed using the Illumina TruSeq RNA Sample Preparation kit following manufacturer's instructions. Briefly, RNA was subjected to QC, chemically fragmented, and reverse transcription performed using random primers. cDNA was converted to double stranded cDNA and indexed adapters ligated. The final adapter-ligated cDNA was enriched through 10 cycles of PCR, and cleaned before being quantified by qRT-PCR. Libraries were pooled and sequenced on an Illumina HiSeq 2500 system, generating an average of 59.6 +/- 8.7 million paired reads per sample of length 100 bp. Unique barcodes were used to demultiplex the samples.

Reads were aligned to the mouse genome (Mus_musculus.GRCm38.68 from Ensembl) using TopHat-2.0.3, sam files produced using SAMtools 0.1.18, and gene counts generated using HTSeq 0.5.3p9 using Mus_musculus.GRCm38.68.gtf. Potential duplicate reads were not removed.

**Data normalisation, correction and calculation of mRNA half lives**

Raw reads for each transcript were normalised to transcript length and to account for pool-specific reaction efficiency. *Capzb* was selected as normaliser due to its ubiquitous expression and long mRNA half-life.

*Raw reads/(gene length x Capzb reads) = Transcript abundance*

Transcript abundance was corrected to account for contamination from the opposing tissue layer. This was done according to expression level in the opposing layer to avoid over correcting of genes not expressed, or under correcting of highly expressed genes.

To correct each epidermal sample’s transcript abundance for dermal contamination:

*Epidermis Col1A1 transcript abundance* */littermate dermis Col1A1 transcript abundance* *= extent of dermal contamination*

*Epidermis transcript abundance* *– (littermate dermis transcript abundance* *x extent of dermal contamination) = Corrected transcript abundance*

To correct each dermal sample’s transcript abundance for epidermal contamination:

*Dermis Krt5 transcript abundance /littermate epidermis Krt5 transcript abundance = extent of epidermal contamination*

*Dermal transcript abundance – (littermate epidermis transcript abundance x extent of epidermal contamination) = Corrected transcript abundance*

Expression levels for each timepoint were calculated by normalising t_x_ corrected transcript abundance to the corresponding t_0_ corrected transcript abundance, and setting the 0 minute timepoint expression level to 1:

*tx corrected transcript abundance / t0 corrected transcript abundance = timepoint expression level*

Decay constants (k) were calculated using Microsoft Excel by determining the slope of the exponential curve fitted to the timepoint expression levels and setting the *y* intercept to 1. This was achieved by taking the natural log of the data points, calculating line statistics using the LINEST function, and using the INDEX function to return the slope. The FALSE condition was included to set the *y* intercept to 0 (the natural log of 1). The following formula was used, where known *y*s are the timepoint expression levels, and known *x*s the timepoints in minutes.

k=INDEX(LINEST(LN(known *ys*),known *xs*,FALSE),1)

Half-lives were calculated using the equation LN(2)/k.

**qRT-PCR validation of mRNA half lives**

E13.5 dorsal skin explants were treated in culture with Actinomycin D for 30, 60 or 120 minutes as previously described. Samples were an independent set from those used in the RNA sequencing experiment. RNA was extracted as described and cDNA synthesised from 200 ng RNA. qRT-PCR was performed for a selection of epidermal and dermal transcripts, and normalised to *Capzb*. Relative expression levels for each sample were calculated by normalising t_x_ expression levels to their corresponding t_0_ halves, and setting the t_0_ relative expression levels to 1. Relative expression levels for each timepoint were the means of the results from at least 3 separate skin explants. Decay constants were determined from the slope of the exponential curve fitted through the data points on an Excel-generated chart.
